# OmpA protein sequence-based typing and virulence-associated gene profiles of *Pasteurella multocida* isolates associated with bovine haemorrhagic septicaemia and porcine pneumonic pasteurellosis in Thailand

**DOI:** 10.1186/s12917-017-1157-6

**Published:** 2017-08-16

**Authors:** Teerasak E-kobon, Ratiporn Leeanan, Saengtian Pannoi, Pornchai Anuntasomboon, Pacharee Thongkamkoon, Arinthip Thamchaipenet

**Affiliations:** 1 Department of Genetics, Faculty of Science, Kasetsart University, 50, Ngam Wong Wan Rd, Lad Yao, Chatuchak, Bangkok, 10900 Thailand; 2 Bioinformatics and Systems Biology Unit, Computational Biomodelling Laboratory for Agricultural Science and Technology (CBLAST), Faculty of Science, Kasetsart University, 50 Ngam Wong Wan Rd, Lat Yao, Chatuchak, Bangkok, 10900 Thailand; 3 Laboratory of Bacteriology and Mycology, National Institute of Animal Health, Department of Livestock, Ministry of Agriculture and Cooperatives, 50/2 Kasetklang, Lad Yao, Chatuchak, Bangkok, 10900 Thailand

**Keywords:** *Pasteurella multocida*, *ompA*, Pneumonic pasteurellosis, Haemorrhagic septicaemia, Virulence-associated genes, Molecular evolution, Molecular typing

## Abstract

**Background:**

*Pasteurella multocida* is a Gram-negative bacterium that causes economically significant infections of a broad range of animal species. Pneumonic and septicaemic pasteurellosis caused by this bacterium remain important problems in pigs, cattle, and water buffaloes in Thailand. The aim of this study was to characterise the virulence-associated gene profiles and to develop an OmpA molecular typing scheme for classifying 191 bovine and porcine isolates of *P. multocida* collected between 1989 and 2012 in Thailand using polymerase chain reactions (PCRs), nucleotide sequencing, and sequence and structural bioinformatics analyses.

**Results:**

PCR screening successfully characterised the profiles of 25 virulence-associated genes in all isolates. The gene profiles separated these isolates into bovine and porcine clusters based on eight genes (*hgbB*, *hsf1*, *tadD*, *nanH*, *pfhA*, *plpE*, *pmHAS*, and *tbpA*). Phylogenetic analyses of the nucleotide and protein sequences corresponding to the *ompA* gene, which encodes a major outer membrane surface protein, showed two major bovine and porcine clusters. Structural prediction and analysis of the dN/dS ratio revealed four hypervariable extracellular loops of the OmpA transmembrane domains. These four loops were used to develop an OmpA typing scheme. This scheme classified 186 isolates into five major loop sequence types (LST8, LST12, LST15, LST18, and LST19), consistent with the phylogenetic results. The loop regions of the bovine isolates were predicted to be more antigenic than those of the porcine isolates. Thus, molecular evolution of the OmpA proteins could be used to classify *P. multocida* isolates into different capsular types, host types, and, possibly, pathogenicity levels.

**Conclusions:**

Together with the virulence-associated gene profiles, the typing reported in this work provides a better understanding of *P. multocida* virulence. Effective monitoring and potential strain-specific subunit vaccines could be developed based on these loop oligopeptides.

**Electronic supplementary material:**

The online version of this article (doi:10.1186/s12917-017-1157-6) contains supplementary material, which is available to authorized users.

## Background

*Pasteurella multocida* is a facultative anaerobic Gram-negative bacterium responsible for economically significant infections in a broad range of animal species [1]. *P. multocida* commonly presents as a commensal bacterium in the respiratory tract but can become a primary or secondary pathogen depending on health status, animal species, climatic conditions, and poor husbandry practices [1, 2]. The organism causes a variety of respiratory diseases, including pneumonic pasteurellosis of ruminants and pigs, porcine progressive atrophic rhinitis (PAR), avian cholera, bovine haemorrhagic septicaemia (HS), snuffles in lagomorphs, and human infections via bites or scratches from carnivores [2]. Pneumonic and septicaemic pasteurellosis remain important problems in pigs, cattle, and water buffaloes worldwide. The disease severity varies from acute to subacute to chronic in different animal hosts [3] and depending on the bacterial serotype [4]. Generally, this organism can be classified into five capsular serotypes (A, B, D, E, and F) and 16 lipopolysaccharide (LPS) serotypes (1–16) [3]. Associations of certain serotypes of *P. multocida* with specific disease symptoms or geographical areas have been systematically shown by Davies et al. [5–9]. How these serotypic preferences and host predilections contribute to the virulence of *P. multocida* is not fully understood.

Recently, several comparative genomic studies have revealed different genes and genetic elements between virulent strains of *P. multocida* and avirulent strains [10–12]. The pathogenicity and virulence of *P. multocida* involve multiple genes related to the bacterial capsule, endotoxins (LPS), dermonecrotic exotoxin or *P. multocida* toxin, phages, plasmids, type IV pili, adhesins, extracellular enzymes, and outer membrane proteins (OMPs) [2, 13, 14]. Several genes encoding these features of *P. multocida*, including *exbB*, *exbD*, *fimA*, *fur*, *hgbA*, *hgbB*, *hsf1*, *hsf2*, *nanB*, *nanH*, *oma87*, *ompA*, *ompH*, *pfhA*, *plpB*, *plpE*, *pmHAS*, *psl*, *ptfA*, *sodA*, *sodC*, *tadD*, *tbpA*, *tonB*, and *toxA*, have been used in molecular typing studies to define virulence-associated gene profiles. The products of the *exbB*, *exbD*, *fur*, and *tonB* genes are needed for energy transfer from the inner membrane to the outer membrane iron transporters [15]. The *hgbAB* and *tbpA* genes encode outer membrane haemoglobin and transferrin transporters that are tightly regulated by the product of the *fur* gene [16]. The *fimA*, *hsf1, hsf2*, *pfhA*, *ptfA*, and *tadD* genes encode adhesion and colonisation factors that allow *P. multocida* to colonise host mucosal membranes [2, 17–19]. The products of the *nanB*, *nanH*, *ompH*, *plpB*, and *psl* genes are OMPs involved in nutrient acquisition and transport. The sialidase proteins NanB and NanH cleave sialic acids from the host membrane for use as nutrients and in modifying the cell envelope to evade the host immune system [2, 20]. OmpH, a porin, is a general transporter that facilitates the diffusion of various molecules [21]. The superoxide dismutases *sodA* and *sodC* are important in the oxidative stress response and in protection against oxidative stress [22]. The *ompA* and *oma87* genes are required for the biosynthesis and integrity of the outer membrane, the outermost surface of the bacterium that interfaces with the external environment [23]. The highly abundant OmpA protein possesses a C-terminal globular domain that interacts with the peptidoglycan layer of the cell wall, providing stability and integrity to the outer membrane. The OmpA protein is also involved in adherence to host cells through heparin and fibronectin binding [24]. The outer membrane lipoproteins PlpB, PlpE and Psl (P6-like) may stimulate immune protection against the bacterium in animal hosts, but their precise functions remain unclear [25–27]. The *toxA* gene encodes a dermonecrotic toxin (DMT) that blocks chemotaxis-induced migration of dendritic cells to lymph nodes and restricts the progress of the adaptive immune response [2]. Some of these virulence-associated genes appear only in virulent strains of the bacterium and are highly correlated with disease severity. Others may also be present in non-pathogenic strains but are highly expressed only in virulent strains in vivo, as shown by Li et al. [28].

The existing evidence suggests that the virulence-associated genes described above may be required for pathogenic strains of *P. multocida* to survive under host conditions. Several studies have screened these genes in a range of *P. multocida* isolates in attempts to identify their involvement in virulence and host specificity [29–33]. These studies revealed the prevalence of the *toxA* gene in *P. multocida* strains (mainly capsular type D) associated with porcine PAR and frequent observation of the *tbpA* gene in the strains that affect cattle. Many of these genes, particularly the OMP-encoding genes, have been widely studied, and some have been used as the basis for the development of vaccines that can heterologously protect against infections by multiple strains of *P. multocida* [13, 14, 34–39]. This evidence has led to the hypothesis that molecular variations within the same virulence-associated genes may affect host specificity and virulence. Verma et al. found seven classes of *ompA* alleles in 46 isolates of *P. multocida* [40]*.* Associations of these *ompA* alleles with capsular type and disease status have been proposed. Another recent study identified associations between *ompH* alleles and the capsular types of 83 isolates of *P. multocida* and found variable *ompH* alleles within capsular type A isolates [41]. This information is important for detailed typing of virulence isolates and will be helpful in the monitoring of *P. multocida* infections. However, the prevalence of specific virulence-associated genotypes and OmpA types in pigs, cattle, and water buffaloes in Thailand has not been determined. Therefore, this study aimed to characterise the virulence-associated gene profiles of 191 bovine and porcine *P. multocida* isolates collected in Thailand over a 24-year period and to develop a protein-sequence-based OmpA typing scheme using molecular genetics and bioinformatics techniques. The results of this study provide an alternative molecular typing of *P. multocida* isolates and serve as a basis for the development of OmpA type-specific vaccines.

## Results

**Fig. 1:**
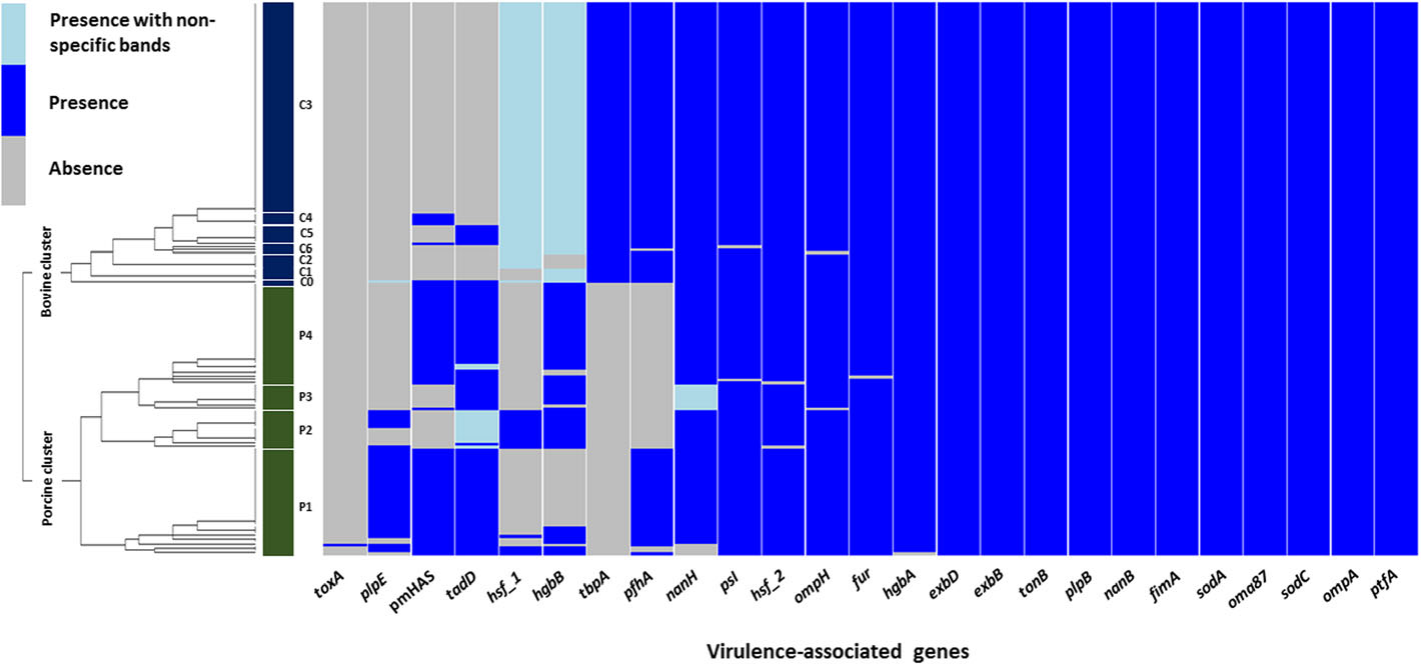
Hierarchical clustering results of 25 virulence-associated gene profiles. The dendrogram shows the hierarchical clustering results of 25 virulence-associated gene profiles (represented as a three-colour heatmap) obtained from 191 isolates of *P. multocida* associated with diseased pigs, cattle, and water buffaloes in Thailand. The letter C is used to indicate the clusters of cattle and water buffaloes (C1, C2, C3, C4, C5, and C6), which are shown in dark blue; P indicates the pig clusters (P1, P2, P3, and P4), which are shown in green

Clustering of 25 virulence gene profiles of 191 bovine and porcine isolates of *P. multocida* revealed two major clusters: bovine and porcine (Fig. 1). Ninety-nine percent of the isolates had the profiles of 17 virulence genes in common (the presence of *exbB*, *exbD*, *fimA*, *fur*, *hgbA*, *hsf2*, *nanB*, *oma87*, *ompA*, *ompH*, *plpB*, *psl*, *ptfA*, *sodA*, *sodC*, and *tonB* and the nearly complete absence of *toxA*). The dendrogram in Fig. 1 shows that the profiles of eight virulence genes (*hgbB*, *hsf1*, *tadD*, *nanH*, *pfhA*, *plpE*, *pmHAS*, and *tbpA*) could be used to separate the isolates into two major clusters. The bovine cluster (shown in *dark blue* in Fig. 1) included five genes (*hsf1*, *hgbB*, *nanH*, *pfhA*, and *toxA*) that were shared across the seven subgroups. The profiles of the eight virulence genes varied across the four subgroups of the porcine cluster (shown in *green* in Fig. 1). The profiles of the Thai bovine isolates of *P. multocida* were less diverse than those of the porcine isolates.

In this study, the bovine isolates (C cluster) were divided into six subgroups — C1, C2, C3, C4, C5, and C6 — based on the profiles of the genes *hgbB*, *hsf1*, *tadD*, and *pmHAS*. The third subgroup, C3, accounted for 76% of the bovine isolates. There was no clear separation between the isolates obtained from cattle and those obtained from water buffaloes or between samples collected in different years. The subgroups C1, C2, C3, C6, and C0 displayed similar virulence gene profiles lacking *pmHAS*, *plpE*, and *tadD* (Fig. 1). The subgroups C4 and C5 additionally displayed the presence of the *pmHAS* and *tadD* genes, respectively. Unlike the porcine cluster, almost all of the bovine isolates possessed the *hsf1*, *hgbB*, *pfhA*, and *tbpA* genes; thus, these genes could be key to the success of these strains in causing acute HS.

The second cluster of porcine isolates was divided into four subgroups: P1, P2, P3, and P4 (Fig. 1). Subgroup P4 (40%), followed by subgroup P1 (37%), included the highest number of porcine isolates. Most isolates of subgroup P1 had the *nanH*, *pfhA*, *plpE*, *pmHAS*, and *tadD* genes in common and showed complete loss of *tbpA*. The *pfhA* gene was only present in subgroup P1, whereas *hsf1* and *hgbB* were present in some isolates of this subgroup. All members of the P1 subgroup belonged to capsular type A, including two bovine type A isolates: 1C and 12C. Similarly, nearly all members of subgroup P4, with the exception of the 104P strain, also belonged to capsular type A. These strains had the *nanH*, *pmHAS*, *hgbB*, and *tadD* genes in common. Two other subgroups of the porcine cluster, subgroups P2 and P3, were isolates of capsular type D. All isolates of subgroup P2 showed the presence of *hsf1*, *nanH*, *hgbB*, and *tadD* genes together with the lack of *pfhA*, *pmHAS*, and *tbpA* genes. Some isolates in this subgroup had the *plpE* gene. The third subgroup, P3, only shared the *tadD*, *hgbB*, and *nanH* genes; the other five genes were absent. These results demonstrate the presence of variable virulence gene-associated profiles in porcine isolates of *P. multocida* in Thailand.

**Fig. 2:**
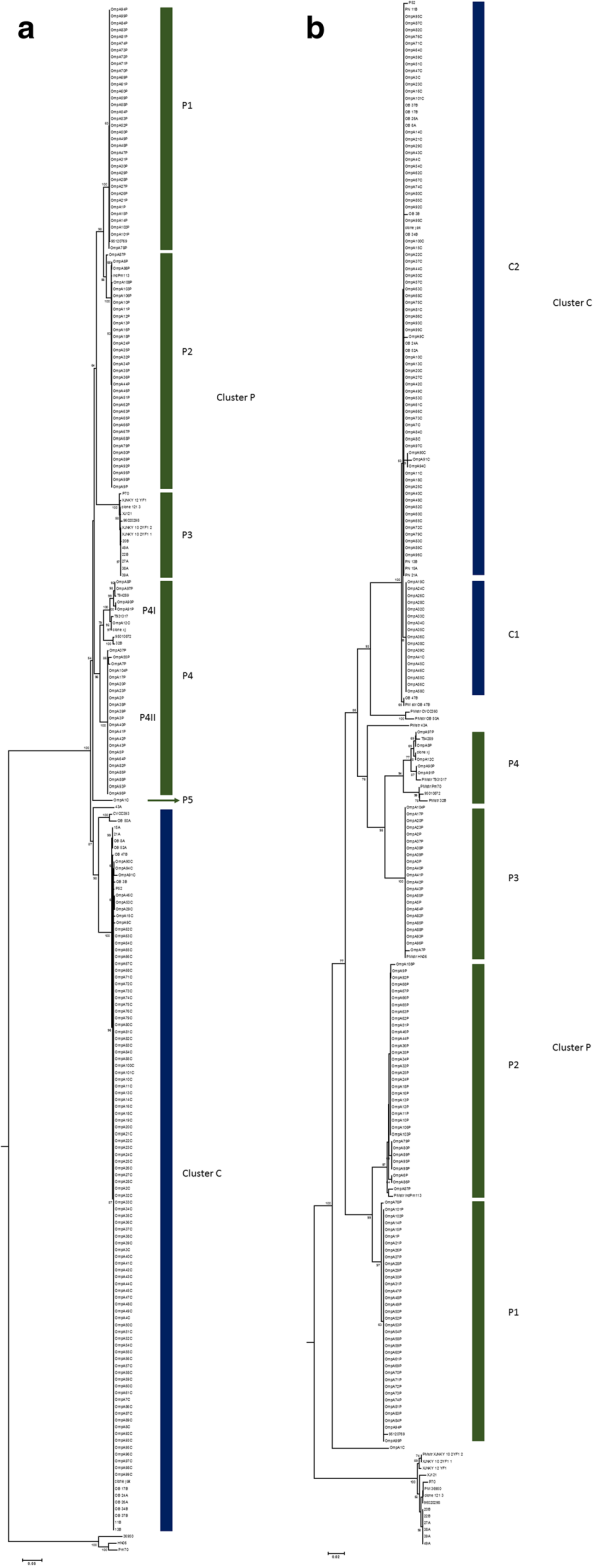
Phylogenetic trees of partial *ompA* genes and proteins. Molecular evolutionary history of partial *ompA* genes (**a**) and proteins (**b**) obtained from 186 *P. multocida* isolates associated with diseased cattle, water buffaloes, and pigs in Thailand in comparison with 41 available *ompA* genes and proteins from the NCBI database. The phylogenetic relationships were inferred using the neighbour-joining method. Evolutionary analyses were conducted in MEGA 6. The bovine clusters (C1 and C2) are labelled in the dark blue bars, and the porcine clusters (P1, P2, P3, and P4) are labelled in the green bars. The P4 subcluster consists of the P4I and P4II groups. Details regarding each cluster are presented in Additional file 2: Table S2

The *ompA* gene was successfully amplified and sequenced from 186 isolates of *P. multocida* in this study. The obtained nucleotide sequences were trimmed to a length of 940 nucleotides and were confirmed to correspond to the *ompA* gene. The obtained sequences covered nearly the full length of the transmembrane domain of this gene. Forty-one available *ompA* nucleotide sequences were also downloaded from the NCBI nucleotide database and compared with the sequences identified in this study. After multiple sequence alignment and phylogenetic construction of 227 DNA sequences, the phylogenetic tree of the *ompA* gene of *P. multocida* (Fig. 2a and Additional file 1: Table S1) clearly showed two major clusters of the bovine (cluster C) and porcine (cluster P) isolates, consistent with the clustering of the virulence-associated genes shown in Fig. 1. The bovine cluster consisted of a single large group of 97 bovine isolates (79 Thai isolates and 18 database strains) of capsular type B. The second porcine cluster consisted of five subgroups: P1, P2, P3, P4, and P5. Sixty-five of the porcine isolates were almost equally clustered in subgroups P1 and P2. Subgroup P3 members (13 strains) included only the samples in the NCBI database. Thirty isolates of subgroup P4 were further divided into subgroups P4I (9 isolates) and P4II (21 isolates). With the exception of a single capsular type D isolate, 87P (subgroup P2), the isolates in subgroups P1, P2, P4I, and P5 were capsular type A. All isolates in the P4II subgroup were capsular type D. These results demonstrate the presence of molecular differences in the *ompA* genes of the two capsular types of porcine *P. multocida* isolates. Interestingly, two bovine isolates of capsular type A (1C and 12C) also grouped within the porcine cluster (subgroups P5 and P4I).

**Fig. 3:**
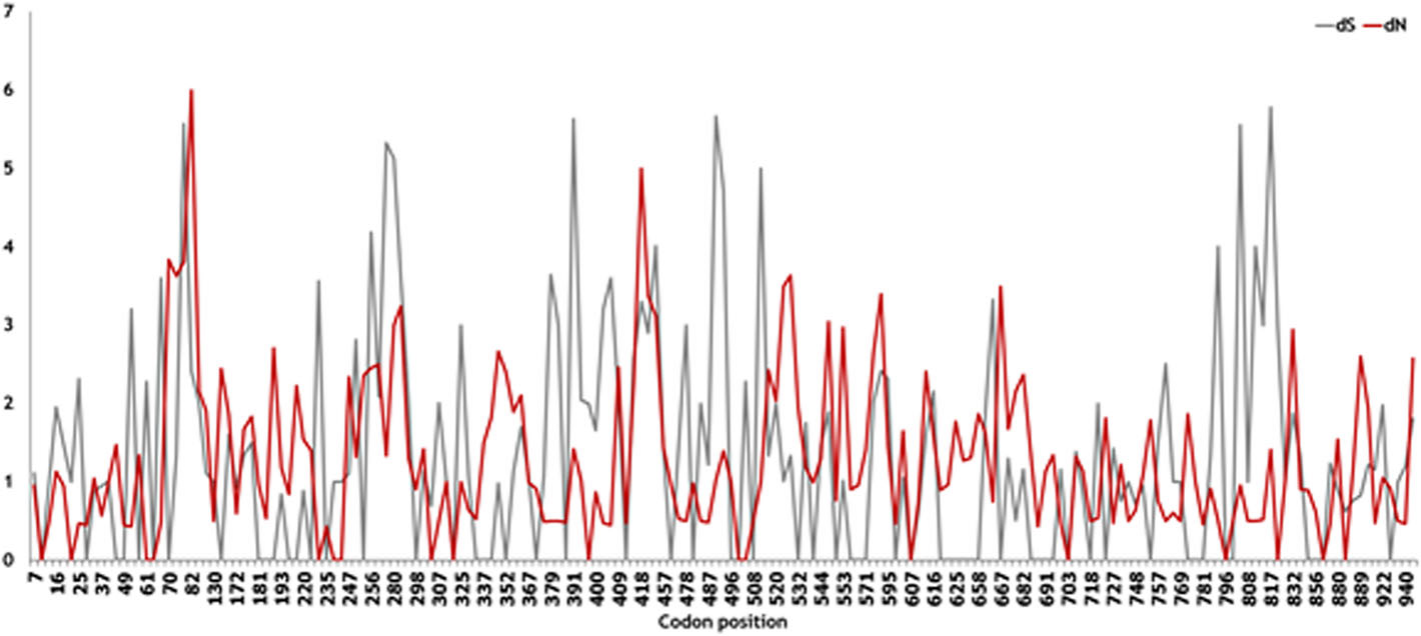
Codon-based dN/dS ratio of the OmpA protein. Calculation of the dN and dS values of each codon of the *ompA* gene of *P. multocida* as a basis for examining the effects of natural selection at each codon position. The x-axis shows the codon positions; the y-axis shows the dN and dS values. The analysis was conducted using the MEGA 6 program

Analysis of nucleotide substitutions within the *ompA* gene was conducted on 95 unique *ompA* sequences, returning a dN of 1.3667, a dS of 1.0341, and a dN/dS ratio of 0.8751. The dN and dS of each codon position in 227 nucleotide sequences of the *ompA* genes analysed in this study were compared (Fig. 3). The results showed that selection pressure had operated unequally on different regions of the *ompA* genes of *P. multocida*. Codon positions 61–220 accumulated more nonsynonymous than synonymous changes, as did positions 409–457. Codon positions 247–325 displayed more synonymous changes, as did positions 460–508 and 781–820, whereas positions 544–780 showed a counterbalance between synonymous and nonsynonymous substitutions.

**Fig. 4:**
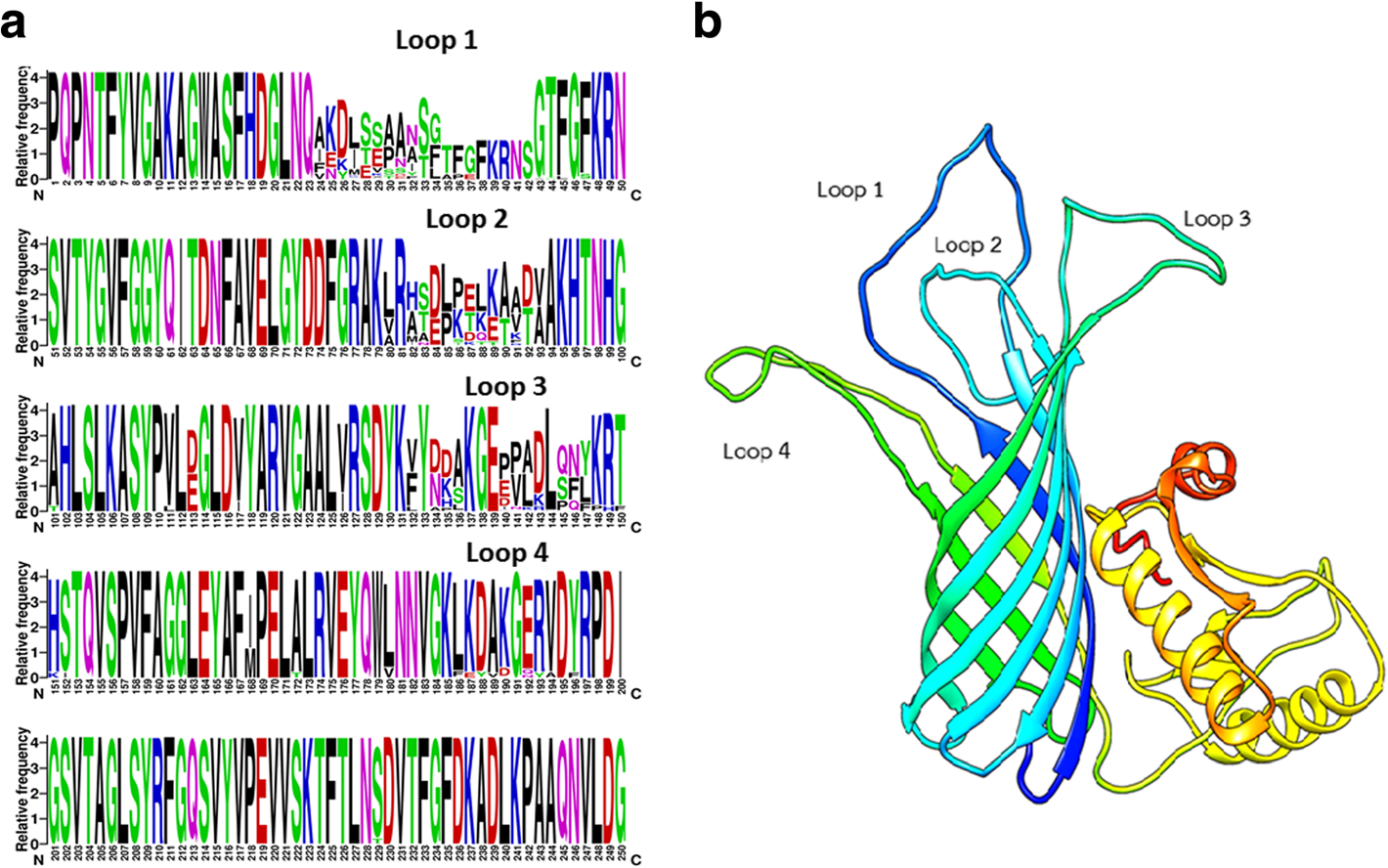
Alignment of the OmpA proteins in association with the predicted OmpA structure. Logo plot summarising the multiple sequence alignment of the partial OmpA sequences of 186 bovine and porcine isolates of *P. multocida* in Thailand using the WebLogo program (**a**). The height of the stack indicates the sequence conservation level at that position. The symbol height also indicates the relative frequency of amino acids at that position. The alignment was mapped onto the homology-predicted OmpA structure (**b**) using the Chimera program to identify substructures of the OmpA protein. These substructures were mapped back onto the alignments as loop 1, loop 2, loop 3, and loop 4. The ompA structures were predicted based on the templates of the OmpA-like domain from *Acinetobacter baumannii* (4g4x_A) and the OmpA transmembrane domain from *Escherichia coli* (1qjp) using the HHPRED and MODELLER programs. The domain shown in yellow is the partial C-terminal peptidoglycan-binding domain, and the domain shown in green is the transmembrane ß-barrel N-terminal domain. The model was visualised using the Chimera program

The OmpA proteins encoded by the *ompA* gene sequences in this study contained 314 amino acids. Compared to the amino acid residues in the full-length OmpA proteins in the Uniprot database, 39 amino acid residues were not included in this study. Twenty-two amino acids were missing from the N-terminus, whereas the C-terminus was shortened by 16 amino acids. The sequences of the transmembrane domain were obtained, and the equally trimmed 186 sequences of this domain were aligned and summarised in a logo plot (Fig. 4a), which showed three variable regions at amino acid positions 24–42, 80–93, and 132–147. These regions correlated well with the dN/dS results for regions 60–159, 248–296, and 415–455, respectively (Fig. 3).

Approximately 252 amino acids within the transmembrane domain were compared (Fig. 4a); the resulting protein phylogenetic tree is shown in (Fig. 2b and Additional file 1: Table S1). The tree shows two major clusters of OmpA proteins from the Thai bovine and porcine isolates. The first cluster consists of two subgroups (P1 and P2) of the porcine isolates associated with capsular type A. The second cluster contains a very large subgroup C (C1 and C2) of the bovine isolates of capsular type B and a small subgroup of the porcine (P3 and P4) isolates. The members of subgroup P3 are all porcine isolates associated with capsular type D, whereas the members of subgroup P4 are capsular type A isolates, including the bovine isolate 12C. Although this protein tree differs slightly from the nucleotide tree derived for the *ompA* gene, the distinctions between animal hosts and capsular types are clearly shown.

**Fig. 5:**
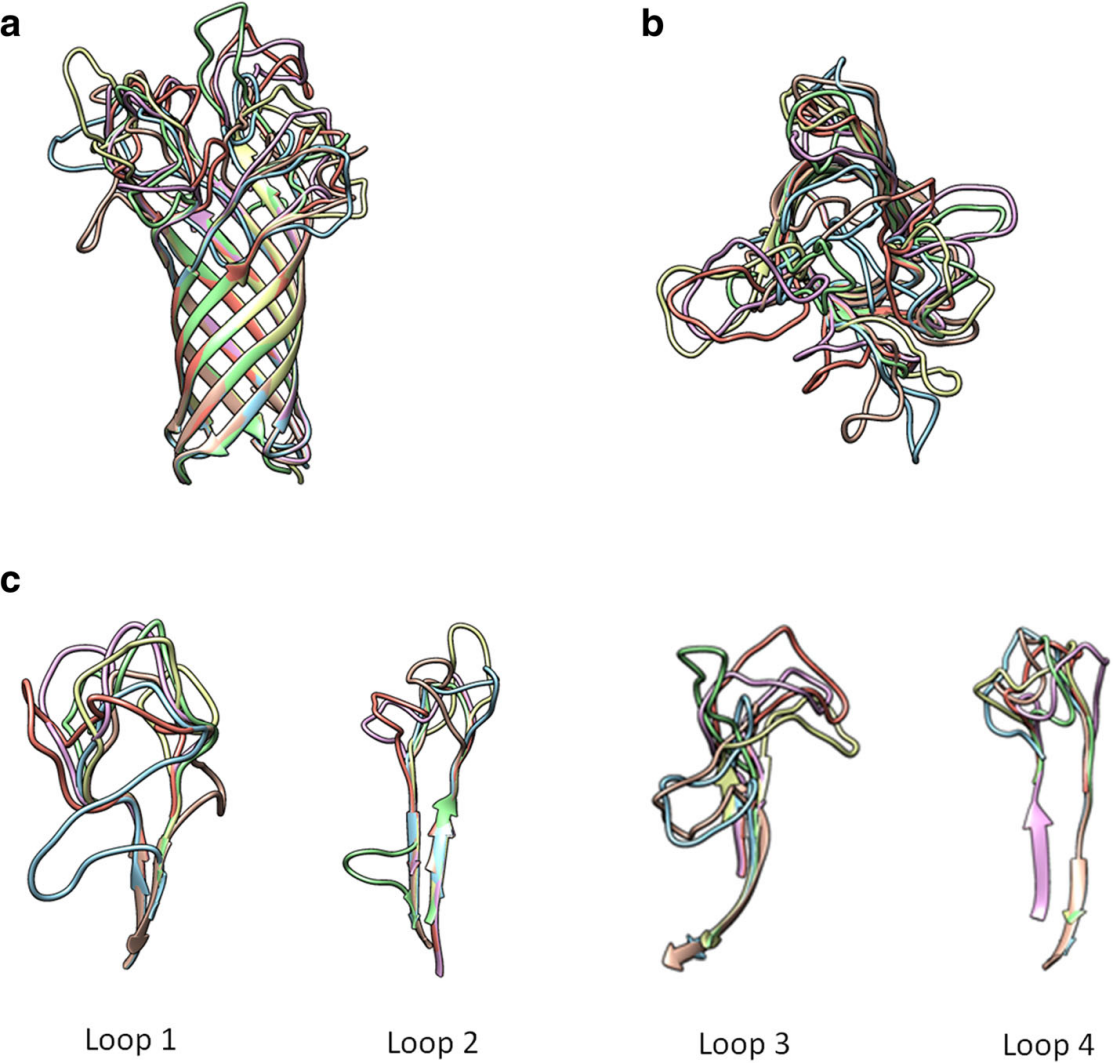
Comparative structures of the OmpA proteins. Comparative structures of the OmpA proteins from six protein clusters (P1, P2, P3, P4, C1, and C2) according to the protein phylogenetic tree (Fig. 2b) of the OmpA proteins of *P. multocida*. A side view of the conserved transmembrane ß-sheets is shown in **a**, and the four hypervariable loops are shown in the top view (**b**). The individual extracellular loops are compared in **c**. The colours represent different proteins: *orange* indicates isolate 21P, *blue* indicates isolate 32P, *green* indicates isolate 42P, *yellow* indicates isolate 91P, *pink* indicates isolate 34C, and *red* indicates isolate 84C

**Fig. 6:**
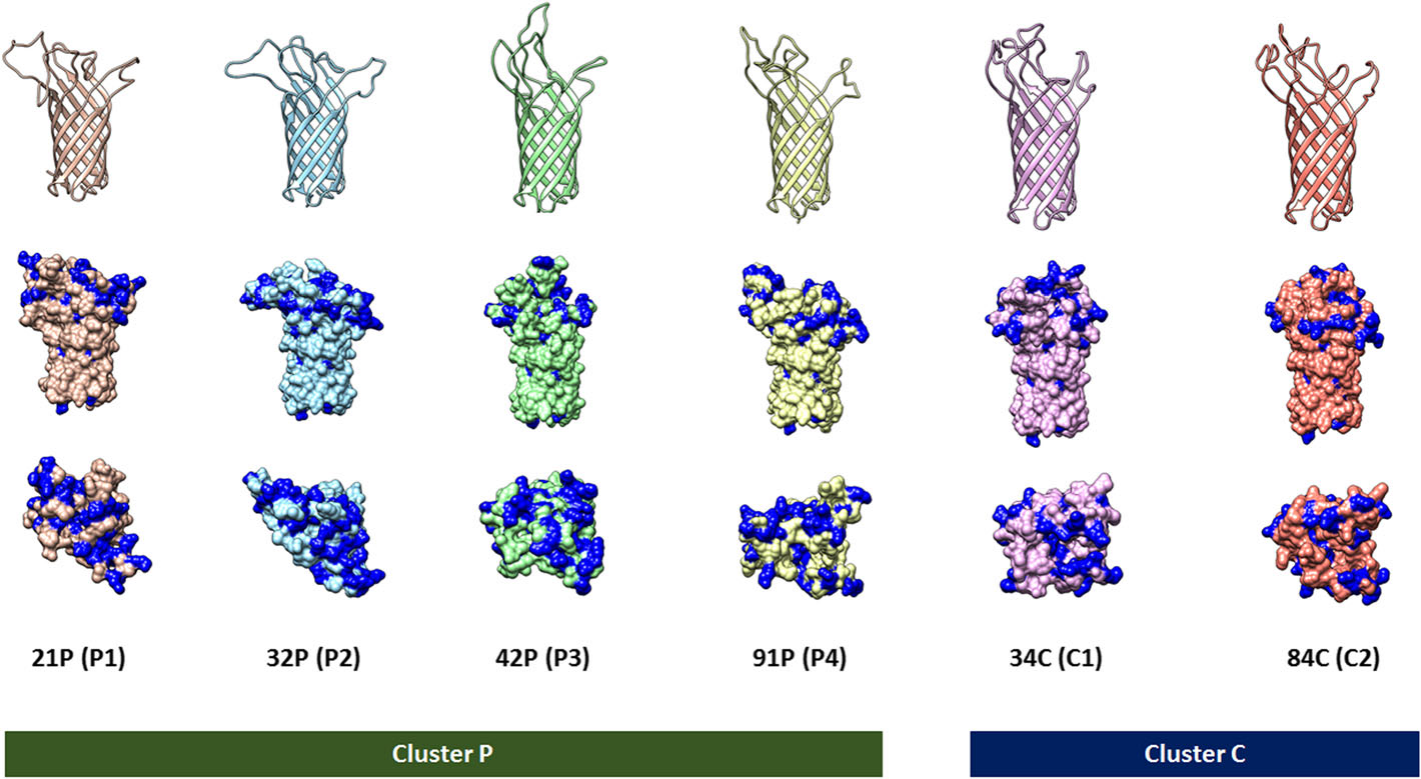
Surface structures of the predicted OmpA proteins. Predicted structures of the OmpA proteins from six major groups (P1, P2, P3, P4, C1, C2) according to the protein tree (Fig. 2b) of bovine and porcine isolates of *P. multocida* in Thailand. These structures are shown in ribbon (*top*) and surface-view (*middle* and *bottom*) formats. In the surface-view format, the residues highlighted in blue represent charged amino acids. Side and top views of the surface-view format are shown in the middle and bottom rows

The multiple aligned OmpA sequences were subjected to structural prediction. The consensus OmpA structure of *P. multocida* strains in Thailand was predicted based on the template structures of the OmpA-like domain from *Acinetobacter baumannii* (4g4x_A) and the OmpA transmembrane domain from *Escherichia coli* (1qjp) (Fig. 4b). The predicted OmpA structure of the Thai isolates of *P. multocida* contained two domains: a globular C-terminal peptidoglycan-binding domain and a transmembrane ß-barrel N-terminal domain. The transmembrane domain consisted of eight antiparallel ß-sheets interspersed with four short periplasmic turns and four long extracellular loops, similar to the OmpA proteins of other bacteria. Six OmpA structures (21P, 32P, 42P, 91P, 34C, and 84C) were predicted as representative of six major OmpA protein clusters (P1, P2, P3, P4, C1, and C2) from the tree shown in Fig. 2b. Superposition of these structures revealed eight conserved transmembrane ß-sheets and four hypervariable loop regions (Fig. 5a and b). The fourth loop was more structurally conserved than the other three loops (Fig. 5c). Individual models of these six OmpA proteins are presented in Fig. 6. These representative structures were classified into two clusters, similar to the protein clustering shown in Fig. 2b. By computing the molecular surface, the extracellular loops of these six proteins were determined to be rich in charged amino acid residues (middle and bottom rows in Fig. 6). Taken together, the data show that the observed variations in the nucleotide sequences of the *ompA* genes contribute to the structures of the hypervariable extracellular loop regions of the OmpA proteins.

The amino acid sequences corresponding to the four hypervariable extracellular loops (L1, L2, L3, and L4) were extracted from the alignment and trimmed to the same length. The first loop (L1) was closest to the N-terminus, whereas the last loop (L4) was close to the C-terminal domain. A difference in at least one amino acid position within the same loop was considered to represent a distinct loop sequence type (LST). This study developed the OmpA protein sequence-based typing scheme using the observed LSTs. Each LST consisted of four loop types: loop 1 sequence type (L1ST), loop 2 sequence type (L2ST), loop 3 sequence type (L3ST), and loop 4 sequence type (L4ST), which was formularised as LST = L1ST: L2ST: L3ST: L4ST. The use of LST molecular typing on these isolates revealed 30 LSTs comprising 21 L1STs, 11 L2STs, 14 L3STs, and 10 L4STs. Fourteen LSTs were found in 186 Thai bovine and porcine isolates; the other 16 LSTs were present only in the database strains. Five LSTs (LST8, LST12, LST15, LST18, and LST19) included 80% of the Thai isolates. The bovine OmpA proteins in subgroup C1 (capsular type B) harboured LST19 (11:5:5:3), whereas subgroup C2 (capsular type B) belonged to the LST8 group (6:5:5:3). The porcine subgroups P1, P2, and P3 had LST12 (8:3:3:3), LST15 (9:7:3:3), and LST18 (10:2:2:3), respectively. Clearly, the porcine strains of capsular types A (subgroups P1 and P2) and D (subgroup P3) could be differentiated based on their LSTs.

To understand the importance of these variations, the four hypervariable loop sequences of the six subgroups (C1, C2, P1, P2, P3, and P4) were subjected to antigenicity prediction. The prediction algorithm was based on the physicochemical properties of experimentally determined antigens. No loops of the porcine isolates of subgroups P1, P2, and P3 displayed antigenic epitopes. Subgroup P4, which contained the bovine isolate 12C and other porcine isolates, displayed a possibly antigenic first loop. Intriguingly, the bovine subgroups C1 and C2 each had three possibly antigenic loops (L1, L2, and L3). This antigenic variation could have resulted from high levels of nonsynonymous substitution within the loop regions.

## Discussion

Analysis of 25 virulence-associated gene profiles identified 16 genes that may have important functions in all 191 isolates of *P. multocida*. The absence of these genes is expected to greatly affect the survival and likely the virulence of *P. multocida*. Some of these genes may also appear in non-pathogenic strains but may be highly expressed in virulent strains under in vivo conditions, as shown by Li et al. [28]. However, the near absence of the *toxA* gene in this study (it appeared in only one porcine isolate) could reflect successful eradication of *toxA*-positive porcine isolates in Thailand due to the heavy use of antibiotics and vaccines in past years. None of the bovine isolates possessed the *toxA* gene. This finding is consistent with the results of a similar study of Indian and South Asian cattle strains associated with HS, in which the infectious bacteria did not carry the *toxA* gene but harboured the *tbpA* gene [42]. Clustering of the 191 isolates analysed, which was based mainly on the profiles of eight genes, showed that the profiles of the Thai bovine isolates of *P. multocida* were less diverse than those of the porcine isolates. The results were supported by pulsed-field gel electrophoresis (PFGE) and multilocus sequence typing (MLST) of eight field isolates and one vaccine strain of *P. multocida* associated with HS cases from Thailand [43]. All isolates shared the same MLST sequence type 122 and the same PFGE pattern, whereas the method used in the current study was able to distinguish additional minor groups of the bovine isolates.

The prevalence rates of *tbpA*, *pfhA*, and *hgbB* genes in the bovine isolates are also consistent with the results of a study of Japanese cattle strains that exhibited associations of *tbpA*, *pfhA*, and *hgbB* genes with strains collected from diseased animals rather than healthy ones [32]. The virulence-gene-associated profiles of the porcine isolates were highly diverse and showed clear separation between isolates of capsular types A and D. These isolates were associated with chronic and subchronic pneumonic pasteurellosis that persisted and multiplied for long periods (several generations) within the host’s respiratory tract, which could explain why more variations accumulated in these porcine isolates.

PCR amplification of virulence-associated genes *P. multocida* typically uses primers that specifically bind the conserved regions of the virulence-associated genes identified in different isolates of *P. multocida*. This strategy provides a rapid broad scanning of these genes across a large number of isolates, but the results of this method do not provide details on how the genes differ. This study selected the *ompA* gene, which encodes a highly abundant cell-surface-adhesive OMP, for further molecular evolutionary studies. The phylogenetic analyses of the *ompA* gene and its encoded protein conducted in this study confirmed that there is a clear separation between a few clusters of the bovine isolates and more diverse porcine clusters and a distinction between capsular types that is consistent with that previously shown by Verma et al. [40]. The results support the idea that low genetic diversity exists in the clonal population of the bovine isolates of capsular type B in Thailand. This conclusion is consistent with previous studies that compared 16S rRNA genes and found a monophyletic relationship and low genetic diversity of bovine strains of *P. multocida* in the UK [9] and India [44]. Interestingly, two bovine isolates of capsular type A (1C and 12C) collected from water buffaloes and cattle in 1989 and 1995 were grouped within the porcine cluster collected between 2005 and 2009, similar to the gene profiles shown in Fig. 1, suggesting that these bovine isolates were transferred from porcine hosts.

The *ompA* genes displayed a dN/dS ratio of less than one, indicating that these genes are under purifying or stabilising selection that does not allow the genes to change drastically over time, consistent with the calculation by Johnson et al. [11]. The selection pressures on different regions of the *ompA* genes of *P. multocida* were also observed to be unequal based on the fact that the multiple aligned OmpA sequences showed the presence of limited variable regions, a characteristic that could have caused the observed clustering in the protein phylogenetic tree. Mapping the aligned amino acid sequences of the transmembrane domain of the OmpA proteins in this study to the predicted OmpA structures clearly confirmed that the four hypervariable extracellular loops have experienced different selection pressures. Three of these loops were predicted to be more antigenic in the bovine cluster than in the porcine cluster, and these three loops contained more charged residues (positive or negative charges) than other loops, which could determine the specificity of adherence of the bacterium to host cells or to extracellular matrix molecules. These findings suggest that these three extracellular loops are potentially important in host adaptation and that they may play roles in the development of acute HS in cattle and water buffaloes. However, these strong antigenic epitopes might not be necessary for the development of porcine chronic pneumonic pasteurellosis; the adherence function of the loop regions of the OmpA proteins would likely be more important for this prolonged disease. Therefore, the amino acid sequences of these four loops were used to develop the LST scheme through which *P. multocida* strains were directly classified based on the specific sequences of the potential virulence-associated regions of their OmpA proteins.

Evidence for an adherence function of the OmpA protein in *P. multocida* was obtained in this study. OmpA of the bovine strain of *P. multocida* serotype A:3 adheres to heparin, heparin sulphate, type IX collagen, and fibronectin in the extracellular matrix layer of Madin-Darby bovine kidney (MDBK) cells [24, 45]. The OmpA protein preferentially bound to the N-terminal heparin-binding domain of fibronectin [45]. In our study, a heparin-binding site that includes a K/R-X-K/R motif was identified in the second extracellular loop (L2) of the protein (Fig. 4a). This second loop was the most enriched in positively charged amino acids (lysine, arginine, and histidine), accounting for an average of 25% of the loop length. Variations in the amino acid sequence of this loop could indicate certain levels of binding optimisation for specific host cell adherence. Recently, Katoch et al. [46] amplified and sequenced the *ompA* genes of four bovine strains of *P. multocida* (two of capsular type A and two of capsular type B). The authors found two different sequence types of the *ompA* genes that were specific to the strains of capsular types A (allele II) and B (allele I). These differences occurred within the four extracellular loop regions. The authors compared the in vitro and in vivo adhesion and invasion capacities of the two groups of strains and found that the capsular type B group with allele I of the *ompA* gene was more invasive than the other group. Unlike other studies, the current study could also differentiate porcine isolates of capsular types A and D, and bovine isolates of capsular type B based on the protein sequence-based LSTs (not the *ompA* alleles), and the differences in the LSTs could be directly linked to the molecular function of the OmpA protein. These assigned LSTs could potentially be used to determine the virulence of *P. multocida* isolates and further classify other *P. multocida* isolates e.g. bovine and porcine isolates of the same capsular type A. However, in vitro and in vivo assays may be required to confirm these findings.

## Conclusions

Molecular variation of the OmpA proteins could be used to classify *P. multocida* isolates into different capsular types, hosts, and, possibly, pathogenicity levels. Taking into account this protein sequence-based typing together with virulence-associated gene profiles will provide a better understanding of *P. multocida* virulence. Effective monitoring and potential strain-specific subunit vaccines could be developed based on these loop oligopeptides.

## Methods

### Bacterial strains

A total of 191 strains of *Pasteurella multocida* were kindly collected and provided by the National Institute of Animal Health, Department of Livestock, Ministry of Agriculture and Cooperatives, Thailand. These isolates were obtained from clinical cases of pigs, cattle, and water buffaloes in Thailand from 1989 to 2012 (Additional file 2: Table S2). Ninety-two of these isolates were from pneumonic pasteurellosis (70 isolates of capsular type A and 22 isolates of type D) in pigs; the others were HS cases (96 isolates of capsular type B and three isolates of type A) in cattle and water buffaloes. Most of the isolates were collected from lungs (81 samples); some were from hearts (11), livers (9), kidneys (3), lymph nodes (3), tonsils (2), blood (1), and brain (1). However, disease information of 75 isolates was unavailable due to the loss of old data records. All samples were stored at −80 °C. The isolates were plated on blood agar supplemented with 5% inactivated sheep blood and incubated at 37 °C for 24 h.

### Genomic DNA extraction from *P. multocida* isolates

Genomic DNA of each sample was prepared by heat treatment. An aliquot of 1 ml of overnight culture in brain-heart infusion broth (BHIB, Oxoid) was centrifuged at 13,000 xg for 5 min and washed in phosphate-buffered saline (PBS). The pellet was resuspended in DNase- and RNase-free distilled water and heated at 100 °C for 5 min. After final centrifugation at the same speed, the supernatant was used for PCR.

### Selection of virulence-associated genes of *P. multocida*

Twenty-five virulence-associated genes (*exbB*, *exbD*, *fimA*, *fur*, *hgbA*, *hgbB*, *hsf1*, *hsf2*, *nanB*, *nanH*, *oma87*, *ompA*, *ompH*, *pfhA*, *plpB*, *plpE*, *pmHAS*, *psl*, *ptfA*, *sodA*, *sodC*, *tadD*, *tbpA*, *tonB*, and *toxA*) were selected based on previous research [18, 27, 33] and bioinformatics predictions [47]. These genes are involved in nutrient and energy acquisition, cellular transport, adherence, protection from immune attack, biosynthesis of the outer membrane and capsule, and detoxification (Table 1). Primers specific for the candidate genes were selected and modified based on previous work. We confirmed the specificity of the primers using BLASTN searches against an NCBI nucleotide sequence database. Details concerning the selected genes and their corresponding primer pairs are presented in Table 1. The primers were synthesised commercially (Macrogen Co., Ltd., Korea).

**Table 1:** Selected virulence-associated genes and their corresponding primer pairs

No	Gene symbol	Function	Primer name	Primer sequence (5′ ➔ 3′)	Product size (kb)	References
1	*exbB*	Energy transport for iron acquisition	exbB-F exbB-R	TTGGCTTGTGATTGAACGC TGCAGGAATGGCGACTAAA	283	[18]
2	*exbD*	Energy transport for iron acquisition	exbD-F exbD-R	CGTTCTGATTACAGCCTCTT AACGAAATCTTGGAAACTGG	247	[18]
3	*fimA*	Adherence and colonization	fimA-F fimA-R	CCATCGGATCTAAACGACCTA AGTATTAGTTCCTGCGGGTG	806	[18]
4	*fur*	Iron-dependent regulation	fur-F fur-R	GTTTACCGTGTATTAGACCA CATTACTACATTTGCCATAC	244	[18]
5	*hgbA*	Iron acquisition	hgbA-F hgbA-R	TCAACGGCAGATAATCAGGG GCGGGAATGCTGAAGATAAG	268	[18]
6	*hgbB*	Iron acquisition	hgbB-F hgbB-R	ACCGCGTTGGAATTATGATTG CATTGAGTACGGCTTGACAT	768	[33]
7	*hsf1*	Adherence and colonization	hsf1-F hsf1-R	TTGAGTCGGCTGTAGAGTTCG ACTCTTTAGCAGTGGGGACAACCTC	654	[18]
8	*hsf2*	Adherence and colonization	hsf2-F hsf2-R	ACCGCAACCATGCTCTTAC TGACTGACATCGGCGGTAC	430	[18]
9	*nanB*	Nutrition acquisition	nanB-F nanB-R	GTCCTATAAAGTGACGCCGA ACAGCAAAGGAAGACTGTCC	586	[33]
10	*nanH*	Nutrition acquisition	nanH-F nanH-R	GTGGGAACGGGAATTGTGA ACATGCCAAGTTTGCCCTA	287	[18]
11	*oma87*	Outer membrane protein assembly and insertion	oma87-F oma87-R	GGCAGCGAGCAACAGATAACG TGTTCGTCAAATGTCGGGTGA	833	[18]
12	*ompA*	Outer membrane biosynthesis and integrity	ompA-F ompA-R	CGCATAGCACTCAAGTTTCTCC CATAAACAGATTGACCGAAACG	202	[18]
13	*ompH*	Outer membrane general transport	ompH-F ompH-R	CGCGTATGAAGGTTTAGGT TTTAGATTGTGCGTAGTCAAC	452	[18, 33]
14	*pfhA*	Adherence and colonization	pfhA-F pfhA-R	AGCTGATCAAGTGGTGAAC TGGTACATTGGTGAATGCTG	256	[33]
15	*plpB*	Amino acid transport	plpB-F plpB-R	TTTGGTGGTGCGTATGTCTTCT AGTCACTTTAGATTGTGCGTAG	531	[18]
16	*pmHAS*	Capsule biosynthesis	pmHAS-F pmHAS-R	TCAATGTTTGCGATAGTCCGTTAG TGGCGAATGATCGGTGATAGA	430	[18]
17	*psl*	Outer membrane biosynthesis and integrity	psl-F psl-R	TCTGGATCCATGAAAAAACTAACTAAAGTA AAGGATCCTTAGTATGCTAACACAGGACGACG	400	[33]
18	*ptfA*	Adherence and colonization	ptfA-F ptfA-R	TGTGGAATTCAGCATTTTAGTGTGTC TCATGAATTCTTATGCGCAAAATCCT GCTGG	500	[18, 33]
19	*sodA*	Detoxification of radicals	sodA-F soda-R	TACCAGAATTAGGCTACGC GAAACGGGTTGCTGCCGCT	263	[33]
20	*sodC*	Detoxification of radicals	sodC-F sodC-R	AGTTAGTAGCGGGGTTGGCA TGGTGCTGGGTGATCATCATG	237	[33]
21	*tadD*	Adherence and colonization	tadD-F tadD-R	TCTACCCATTCTCAGCAAGGC ATCATTTCGGGCATTCACC	418	[18]
22	*tbpA*	Iron acquisition	tbpA-F tbpA-R	TGCGACAACGGAAATTTCCTC GGACAGTGCATATAACTTGTTTACTA	808	[33]
23	*tonB*	Energy transport for iron acquisition	tonB-F tonB-R	CGACGGTGAAACCTGAGCCA CCGAGCGATAAGCATTGACT	261	[18]
24	*toxA*	Exotoxin	toxA-F toxA-R	CTTAGATGAGCGACAAGGTT GGAATGCCACACCTCTATA	864	[33]
25	*plpE*	Highly immunogenic outer membrane lipoprotein	plpE-F plpE-R	CCATGGGCATGAAACAAATCGTTTTAAA CTCGAGTTGTGCTTGGTGACTTTTTTC	1010	[27]

### PCR amplification of 25 virulence-associated genes

Twenty-five pairs of oligonucleotide primers were used to detect 25 virulence-associated genes from 191 isolates of *P. multocida* associated with disease in pigs, cattle, and water buffaloes in triplicate. One microliter of bacterial genomic DNA was used as a template for each 10-μl PCR mixture containing 1 μl of 10X PCR buffer (Vivantis Technologies Sdn. Bhd., Malaysia), 1 μl of 2 mM dNTPs (Vivantis Technologies Sdn. Bhd., Malaysia), 0.3 μl of 50 mM MgCl_2_ (Vivantis Technologies Sdn. Bhd., Malaysia), 0.2 μl of 5 U Taq DNA polymerase (Vivantis Technologies Sdn. Bhd., Malaysia), and 0.2 μl of each of 2 μM forward and reverse primers. To hasten the PCR screening process, six virulence-associated genes were detected in two sets of multiplex PCRs: the first set consisted of *omp87*, *ptfA*, and *sodC*, and the latter set consisted of *fimA*, *nanB*, and *sodA*. Each multiplex PCR mixture contained 1 μl of genomic DNA, 1 μl of 10X PCR buffer, 1 μl of 2 mM dNTPs, 0.3 μl of 50 mM MgCl_2_, 0.2 μl (5 U) of Taq DNA polymerase, and 1 μl of each of 2 μM forward and reverse primers in a volume of 10 μl. A negative control for amplification was generated using an equal volume of distilled water instead of the genomic DNA. The PCRs were conducted in the T100™ thermal cycler (Bio-Rad Laboratories, Inc., US). For all PCRs except the PCR for *ompH*, the following reaction conditions were used: 4 min of initial denaturation at 94 °C; 30 cycles of 30 s denaturation at 94 °C, 30 s annealing at 55 °C, and 1 min extension at 72 °C; and 9 min of final elongation at 72 °C. For the *ompH* PCR, annealing was conducted for 30 s at 57 °C. The amplified products were analysed by electrophoresis on 1.5% agarose gels (Vivantis Technologies Sdn. Bhd., Malaysia), stained with ethidium bromide, and visualised under UV exposure. The appearance of products of the expected sizes was counted as positive identification.

### Construction of virulence-associated gene profiles of *P. multocida*

Twenty-five virulence-associated genes were screened in 92 porcine, 89 bovine, and 10 unknown isolates of *P. multocida* in Thailand using gene-specific PCR. The presence/absence of these 25 genes in each isolate was used to define the virulence-associated gene profile. The profiles were constructed by assigning 0 for the absence of the expected band, 1 for the presence of the expected band, and 2 for the presence of the expected band together with other nonspecific bands. The profiles of all isolates were analysed by hierarchical clustering using a complete linkage method with the dist() and hclust() functions in the R program [48]. A dendrogram and a heatmap were constructed using the gplots package to visualise the clustering results. The virulence profiles were analysed in terms of animal host, capsular type, and available disease information.

### Nucleotide sequence determination of the *ompA* gene

Primers for amplification of the *ompA* gene were synthesised by Macrogen Co., Ltd., Korea. The gene was amplified using Taq DNA polymerase (PrimeSTAR GXL DNA Polymerase, Takara Bio. Inc., Japan) with the forward (OMPA-F, 5′-AGGATCCATGAAAAAAACAGCAATTGCATTGA-3′) and reverse (OMPA-R, 5′-TCTCGAGTTATTTGTTACCTTTAACAGCGATTTC-3′) primers; the sequences of these primers were modified from Gao et al. [49]. The PCRs contained 5 μl of 50 ng/μl genomic DNA, 10 μl of 5X PCR buffer, 5 μl of 2 mM dNTPs, 1 μl (5 U) of Taq DNA polymerase, and 5 μl of each of 2 μM forward and reverse primers in a total volume of 50 μl. The reaction mixtures were incubated for 30 cycles consisting of initial denaturation at 98 °C for 3 min; denaturation at 98 °C for 10 s, annealing at 55 °C for 15 s, extension at 68 °C for 1 min; and final extension at 68 °C for 3 min. The PCR products were purified using a GF-1 Ambiclean kit (Vivantis Technologies Sdn. Bhd., Malaysia), and the cleaned products were subjected to sequencing with both primers using an Applied Biosystems automatic sequencer (ABI 3730XL) (Macrogen Co., Ltd., Korea). The sequence chromatograms were checked for quality, and the *ompA* sequences were confirmed using the BLASTN program.

### Sequence alignment and phylogenetic analysis of the *ompA* gene

The nucleotide sequences of the *ompA* gene were edited, trimmed, and aligned using BioEdit version 7.1.8 [50] and ClustalW in the MEGA 6 program [51]. Noise and low-quality signals were further removed using in-house-written R scripts. Phylogenetic analyses of the edited nucleotide sequences were conducted using the MEGA 6 program. Phylogenetic trees of the *ompA* gene were constructed based on the neighbour-joining model with the p-distance method and a thousand-replicate bootstrap analysis in the MEGA 6 program. The analysis also included the *ompA* nucleotide sequences of *P. multocida* deposited in the NCBI nucleotide database (http://www.ncbi.nlm.nih.gov/genbank/). A phylogenetic tree was constructed, and all isolates were assigned to clusters. The aligned sequences were then analysed using the MEGA 6 and SNAP [52] programs to estimate the numbers of synonymous (dS) and nonsynonymous (dN) substitutions per residue in the gene. The ratio of nonsynonymous to synonymous nucleotide substitutions (dN/dS ratio) is a measure of selection pressure. Maximum likelihood analysis of natural selection was also computed for each codon.

### Protein sequence analysis and structural prediction of the OmpA protein

The nucleotide sequences of the *ompA* gene were translated using the ExPASy translation tool (http://web.expasy.org/translate/). The amino acid sequences were trimmed and edited using in-house-written R scripts and then aligned to identify conserved and variable regions using ClustalW. These regions were summarised using the WebLogo program [53]. A protein phylogenetic tree was constructed based on the neighbour-joining model with the Poisson correction method and a thousand-replicate bootstrap using the MEGA 6 program. The proteins were then assigned to clusters. A representative OmpA protein from an individual cluster in the protein phylogenetic tree was subjected to homology modelling using the HHPred and MODELLER programs [54] and was visualised using the Chimera [55] program. The OmpA structure essentially consists of two domains: a globular C-terminal peptidoglycan-binding domain and a transmembrane ß-barrel N-terminal domain. The transmembrane domain was composed of eight antiparallel ß-sheets interspersed with four short periplasmic turns and four long extracellular loops [56]. The predicted OmpA transmembrane domains were extracted for the next step in the analysis.

### Development of protein-sequence-based OmpA typing schemes

The conserved and variable regions of the candidate OmpA sequences were mapped onto their corresponding predicted structures by structural superimposition and were compared using the Chimera program. Four extracellular loops and transmembrane ß-sheets of the OmpA transmembrane domain were cleaved at equal lengths across all isolates using R scripts. The amino acid sequences of the variable substructures were multiply aligned using ClustalW, and the unique sequences were assigned subsequence types by the R scripts. The protein sequence-based OmpA typing schemes were a combination of subsequence types (X_1_, Y_1_, Z_1_, …), where X_1_, Y_1_, and Z_1_ are subsequence type 1 of substructures X, Y, and Z used to represent each isolate of *P. multocida*. Finally, the sequences of these substructures were submitted for prediction of antigenic peptides by comparison with experimentally known peptide epitopes [57].

## Additional files

Additional file 1: Table S1. Clustering summary of 186 bovine and porcine isolates of *P. multocida* collected from Thailand and 41 strains from the NCBI database. Three clustering methods were shown including nucleotide and protein phylogenetic analyses, and the OmpA protein sequence-based typing using four extracellular loop sequence types (LSTs) of the transmembrane domain. The LST types in the last column were created by the combination of different types of the four extracellular loops 1, 2, 3, and 4. DNA and protein cluster codes correlated with those in Fig. 2a and b. (DOCX 65 kb)

Additional file 2: Table S2. Information of 191 *P. multocida* isolates associated with diseased pigs, cattle and water buffaloes in Thailand collected from 1989 to 2012. These isolates were collected and maintained by the National Institute of Animal Health, Department of Livestock, Ministry of Agriculture and Cooperatives. Each isolate was named with two-letter code. (DOCX 38 kb)

## Abbreviations

HS: Haemorrhagic septicaemia
LST: Loop sequence type
OmpA: Outer membrane protein A
PAR: Progressive atrophic rhinitis
PCR: Polymerase chain reaction
