# Supplementary material for: OmpA protein sequence-based typing and virulence-associated gene profiles of Pasteurella multocida isolates associated with bovine haemorrhagic septicaemia and porcine pneumonic pasteurellosis in Thailand
Source: BMC Vet Res. 2017 Aug 16;13:243. doi: 10.1186/s12917-017-1157-6 (PMC5559837; doi:10.1186/s12917-017-1157-6)
Supplement: Supplementary file 1 — Clustering summary of 186 bovine and porcine isolates of P. multocida collected from Thailand and 41 strains from the NCBI database. Three clustering methods were shown including nucleotide and protein phylogenetic analyses, and the OmpA protein sequence-based typing using four extracellular loop sequence types (LSTs) of the transmembrane domain. The LST types in the last column were created by the combination of different types of the four extracellular loops 1, 2, 3, and 4. DNA and protein cluster codes correlated with those in Fig. 2a and b. (DOCX 65 kb) [file 12917_2017_1157_MOESM1_ESM.docx]

**Additional file 1: Table S1** Clustering summary of 186 bovine and porcine isolates of *P. multocida* collected from Thailand and 41 strains from the NCBI database. Three clustering methods were shown including nucleotide and protein phylogenetic analyses, and the OmpA protein sequence-based typing using four extracellular loop sequence types (LSTs) of the transmembrane domain. The LST types in the last column were created by the combination of different types of the four extracellular loops 1, 2, 3, and 4. DNA and protein cluster codes correlated with those in Figures 2A and 2B.

| **No** | **Strain code** | **Capsular type** | **Animal host** | **DNA cluster** | **Protein cluster** | **Loop 1 type** | **Loop 2 type** | **Loop 3 type** | **Loop 4 type** | **LST type** |
| --- | --- | --- | --- | --- | --- | --- | --- | --- | --- | --- |
| 1 | 1C | A | buffalo | P5 | C0 | 8 | 8 | 6 | 6 | LST14 |
| 2 | 2P | D | pig | P4II | P3 | 10 | 2 | 2 | 3 | LST18 |
| 3 | 3P | D | pig | P4II | P3 | 10 | 2 | 2 | 3 | LST18 |
| 4 | 5P | D | pig | P4II | P3 | 10 | 2 | 2 | 3 | LST18 |
| 5 | 7P | D | pig | P4II | P3 | 10 | 2 | 2 | 3 | LST18 |
| 6 | 17P | D | pig | P4II | P3 | 10 | 2 | 2 | 3 | LST18 |
| 7 | 20P | D | pig | P4II | P3 | 10 | 2 | 2 | 3 | LST18 |
| 8 | 23P | D | pig | P4II | P3 | 10 | 2 | 2 | 3 | LST18 |
| 9 | 37P | D | pig | P4II | P3 | 10 | 2 | 2 | 3 | LST18 |
| 10 | 38P | D | pig | P4II | P3 | 10 | 2 | 2 | 3 | LST18 |
| 11 | 39P | D | pig | P4II | P3 | 10 | 2 | 2 | 3 | LST18 |
| 12 | 40P | D | pig | P4II | P3 | 10 | 2 | 2 | 3 | LST18 |

**Additional Table 1** Continued.

| **No** | **Strain code** | **Capsular type** | **Animal host** | **DNA cluster** | **Protein cluster** | **Loop 1 type** | **Loop 2 type** | **Loop 3 type** | **Loop 4 type** | **LST type** |
| --- | --- | --- | --- | --- | --- | --- | --- | --- | --- | --- |
| 13 | 41P | D | pig | P4II | P3 | 10 | 2 | 2 | 3 | LST18 |
| 14 | 42P | D | pig | P4II | P3 | 10 | 2 | 2 | 3 | LST18 |
| 15 | 43P | D | pig | P4II | P3 | 10 | 2 | 2 | 3 | LST18 |
| 16 | 55P | D | pig | P4II | P3 | 10 | 2 | 2 | 3 | LST18 |
| 17 | 64P | D | pig | P4II | P3 | 10 | 2 | 2 | 3 | LST18 |
| 18 | 82P | D | pig | P4II | P3 | 10 | 2 | 2 | 3 | LST18 |
| 19 | 85P | D | pig | P4II | P3 | 10 | 2 | 2 | 3 | LST18 |
| 20 | 88P | D | pig | P4II | P3 | 10 | 2 | 2 | 3 | LST18 |
| 21 | 93P | D | pig | P4II | P3 | 10 | 2 | 2 | 3 | LST18 |
| 22 | 96P | D | pig | P4II | P3 | 10 | 2 | 2 | 3 | LST18 |
| 23 | 104P | D | pig | P4II | P3 | 10 | 2 | 2 | 3 | LST18 |
| 24 | 8P | A | pig | P4I | P4 | 13 | 4 | 4 | 4 | LST22 |
| 25 | 90P | A | pig | P4I | P4 | 2 | 4 | 4 | 4 | LST3 |
| 26 | 91P | A | pig | P4I | P4 | 2 | 4 | 4 | 4 | LST3 |
| 27 | 97P | A | pig | P4I | P4 | 13 | 4 | 4 | 4 | LST22 |

**Additional Table 1** Continued.

| **No** | **Strain code** | **Capsular type** | **Animal host** | **DNA cluster** | **Protein cluster** | **Loop 1 type** | **Loop 2 type** | **Loop 3 type** | **Loop 4 type** | **LST type** |
| --- | --- | --- | --- | --- | --- | --- | --- | --- | --- | --- |
| 28 | 12C | A | cattle | P4I | P4 | 5 | 4 | 4 | 4 | LST7 |
| 29 | 6P | A | pig | P2 | P2 | 9 | 7 | 7 | 3 | LST16 |
| 30 | 9P | A | pig | P2 | P2 | 9 | 7 | 3 | 3 | LST15 |
| 31 | 10P | A | pig | P2 | P2 | 9 | 7 | 3 | 3 | LST15 |
| 32 | 11P | A | pig | P2 | P2 | 9 | 7 | 3 | 3 | LST15 |
| 33 | 12P | A | pig | P2 | P2 | 9 | 7 | 3 | 3 | LST15 |
| 34 | 13P | A | pig | P2 | P2 | 9 | 7 | 3 | 3 | LST15 |
| 35 | 16P | A | pig | P2 | P2 | 9 | 7 | 3 | 3 | LST15 |
| 36 | 18P | A | pig | P2 | P2 | 9 | 7 | 3 | 3 | LST15 |
| 37 | 24P | A | pig | P2 | P2 | 9 | 7 | 3 | 3 | LST15 |
| 38 | 25P | A | pig | P2 | P2 | 9 | 7 | 3 | 3 | LST15 |
| 39 | 32P | A | pig | P2 | P2 | 9 | 7 | 3 | 3 | LST15 |
| 40 | 34P | A | pig | P2 | P2 | 9 | 7 | 3 | 3 | LST15 |
| 41 | 35P | A | pig | P2 | P2 | 9 | 7 | 3 | 3 | LST15 |
| 42 | 36P | A | pig | P2 | P2 | 9 | 7 | 3 | 3 | LST15 |

**Additional Table 1** Continued.

| **No** | **Strain code** | **Capsular type** | **Animal host** | **DNA cluster** | **Protein cluster** | **Loop 1 type** | **Loop 2 type** | **Loop 3 type** | **Loop 4 type** | **LST type** |
| --- | --- | --- | --- | --- | --- | --- | --- | --- | --- | --- |
| 43 | 44P | A | pig | P2 | P2 | 9 | 7 | 3 | 3 | LST15 |
| 44 | 46P | A | pig | P2 | P2 | 9 | 7 | 3 | 3 | LST15 |
| 45 | 51P | A | pig | P2 | P2 | 9 | 7 | 3 | 3 | LST15 |
| 46 | 62P | A | pig | P2 | P2 | 9 | 7 | 3 | 3 | LST15 |
| 47 | 63P | A | pig | P2 | P2 | 9 | 7 | 3 | 3 | LST15 |
| 48 | 65P | A | pig | P2 | P2 | 9 | 7 | 3 | 3 | LST15 |
| 49 | 66P | A | pig | P2 | P2 | 9 | 7 | 3 | 3 | LST15 |
| 50 | 67P | A | pig | P2 | P2 | 9 | 7 | 3 | 3 | LST15 |
| 51 | 68P | A | pig | P2 | P2 | 9 | 7 | 3 | 3 | LST15 |
| 52 | 79P | A | pig | P2 | P2 | 9 | 7 | 8 | 3 | LST17 |
| 53 | 80P | A | pig | P2 | P2 | 9 | 7 | 8 | 3 | LST17 |
| 54 | 86P | A | pig | P2 | P2 | 9 | 7 | 7 | 3 | LST16 |
| 55 | 87P | D | pig | P2 | P2 | 12 | 7 | 9 | 3 | LST20 |
| 56 | 89P | A | pig | P2 | P2 | 9 | 7 | 8 | 3 | LST17 |
| 57 | 92P | A | pig | P2 | P2 | 9 | 7 | 3 | 3 | LST15 |

**Additional Table 1** Continued.

| **No** | **Strain code** | **Capsular type** | **Animal host** | **DNA cluster** | **Protein cluster** | **Loop 1 type** | **Loop 2 type** | **Loop 3 type** | **Loop 4 type** | **LST type** |
| --- | --- | --- | --- | --- | --- | --- | --- | --- | --- | --- |
| 58 | 95P | A | pig | P2 | P2 | 9 | 7 | 8 | 3 | LST17 |
| 59 | 98P | A | pig | P2 | P2 | 9 | 7 | 8 | 3 | LST17 |
| 60 | 103P | A | pig | P2 | P2 | 9 | 7 | 3 | 3 | LST15 |
| 61 | 106P | A | pig | P2 | P2 | 9 | 7 | 3 | 3 | LST15 |
| 62 | 108P | A | pig | P2 | P2 | 9 | 7 | 3 | 5 | LST15 |
| 63 | 1P | A | pig | P1 | P1 | 8 | 3 | 3 | 3 | LST12 |
| 64 | 14P | A | pig | P1 | P1 | 8 | 3 | 3 | 3 | LST12 |
| 65 | 15P | A | pig | P1 | P1 | 8 | 3 | 3 | 3 | LST12 |
| 66 | 21P | A | pig | P1 | P1 | 8 | 3 | 3 | 3 | LST12 |
| 67 | 26P | A | pig | P1 | P1 | 8 | 3 | 3 | 3 | LST12 |
| 68 | 27P | A | pig | P1 | P1 | 8 | 3 | 3 | 3 | LST12 |
| 69 | 28P | A | pig | P1 | P1 | 8 | 3 | 3 | 3 | LST12 |
| 70 | 29P | A | pig | P1 | P1 | 8 | 3 | 3 | 3 | LST12 |
| 71 | 30P | A | pig | P1 | P1 | 8 | 3 | 3 | 3 | LST12 |
| 72 | 31P | A | pig | P1 | P1 | 8 | 3 | 3 | 3 | LST12 |

**Additional Table 1** Continued.

| **No** | **Strain code** | **Capsular type** | **Animal host** | **DNA cluster** | **Protein cluster** | **Loop 1 type** | **Loop 2 type** | **Loop 3 type** | **Loop 4 type** | **LST type** |
| --- | --- | --- | --- | --- | --- | --- | --- | --- | --- | --- |
| 73 | 47P | A | pig | P1 | P1 | 8 | 3 | 3 | 3 | LST12 |
| 74 | 48P | A | pig | P1 | P1 | 8 | 3 | 3 | 3 | LST12 |
| 75 | 49P | A | pig | P1 | P1 | 8 | 3 | 3 | 3 | LST12 |
| 76 | 50P | A | pig | P1 | P1 | 8 | 3 | 3 | 3 | LST12 |
| 77 | 52P | A | pig | P1 | P1 | 8 | 3 | 3 | 3 | LST12 |
| 78 | 53P | A | pig | P1 | P1 | 8 | 3 | 3 | 3 | LST12 |
| 79 | 54P | A | pig | P1 | P1 | 8 | 3 | 3 | 3 | LST12 |
| 80 | 58P | A | pig | P1 | P1 | 8 | 3 | 3 | 3 | LST12 |
| 81 | 59P | A | pig | P1 | P1 | 8 | 3 | 3 | 3 | LST12 |
| 82 | 60P | A | pig | P1 | P1 | 8 | 3 | 3 | 3 | LST12 |
| 83 | 61P | A | pig | P1 | P1 | 8 | 3 | 3 | 3 | LST12 |
| 84 | 69P | A | pig | P1 | P1 | 8 | 3 | 3 | 3 | LST12 |
| 85 | 70P | A | pig | P1 | P1 | 8 | 3 | 3 | 3 | LST12 |
| 86 | 71P | A | pig | P1 | P1 | 8 | 3 | 3 | 3 | LST12 |
| 87 | 72P | A | pig | P1 | P1 | 8 | 3 | 3 | 3 | LST12 |

**Additional Table 1** Continued.

| **No** | **Strain code** | **Capsular type** | **Animal host** | **DNA cluster** | **Protein cluster** | **Loop 1 type** | **Loop 2 type** | **Loop 3 type** | **Loop 4 type** | **LST type** |
| --- | --- | --- | --- | --- | --- | --- | --- | --- | --- | --- |
| 88 | 73P | A | pig | P1 | P1 | 8 | 3 | 3 | 3 | LST12 |
| 89 | 74P | A | pig | P1 | P1 | 8 | 3 | 3 | 3 | LST12 |
| 90 | 78P | A | pig | P1 | P1 | 8 | 7 | 3 | 3 | LST13 |
| 91 | 81P | A | pig | P1 | P1 | 8 | 3 | 3 | 3 | LST12 |
| 92 | 83P | A | pig | P1 | P1 | 8 | 3 | 3 | 3 | LST12 |
| 93 | 84P | A | pig | P1 | P1 | 8 | 3 | 3 | 3 | LST12 |
| 94 | 94P | A | pig | P1 | P1 | 8 | 3 | 3 | 3 | LST12 |
| 95 | 99P | A | pig | P1 | P1 | 8 | 3 | 3 | 3 | LST12 |
| 96 | 101P | A | pig | P1 | P1 | 8 | 3 | 3 | 3 | LST12 |
| 97 | 102P | A | pig | P1 | P1 | 8 | 3 | 3 | 3 | LST12 |
| 98 | 2C | B | buffalo | B1 | - | 6 | 5 | 5 | 3 | LST8 |
| 99 | 3C | B | buffalo | B1 | C2 | 6 | 5 | 5 | 3 | LST8 |
| 100 | 4C | B | buffalo | B1 | C2 | 6 | 5 | 5 | 3 | LST8 |
| 101 | 7C | B | cattle | B1 | C2 | 6 | 5 | 5 | 3 | LST8 |
| 102 | 8C | B | buffalo | B1 | C2 | 6 | 5 | 5 | 3 | LST8 |

**Additional Table 1** Continued.

| **No** | **Strain code** | **Capsular type** | **Animal host** | **DNA cluster** | **Protein cluster** | **Loop 1 type** | **Loop 2 type** | **Loop 3 type** | **Loop 4 type** | **LST type** |
| --- | --- | --- | --- | --- | --- | --- | --- | --- | --- | --- |
| 103 | 9C | B | cattle | B1 | C2 | 6 | 5 | 5 | 3 | LST8 |
| 104 | 10C | B | buffalo | B1 | C2 | 6 | 5 | 5 | 3 | LST8 |
| 105 | 11C | B | buffalo | B1 | C2 | 6 | 5 | 5 | 3 | LST8 |
| 106 | 13C | B | buffalo | B1 | C2 | 6 | 5 | 5 | 3 | LST8 |
| 107 | 14C | B | buffalo | B1 | C2 | 6 | 5 | 5 | 3 | LST8 |
| 108 | 15C | B | buffalo | B1 | C2 | 6 | 5 | 5 | 3 | LST8 |
| 109 | 16C | B | buffalo | B1 | C2 | 6 | 5 | 5 | 3 | LST8 |
| 110 | 18C | B | buffalo | B1 | C2 | 6 | 5 | 5 | 3 | LST8 |
| 111 | 19C | B | buffalo | B1 | C1 | 11 | 5 | 5 | 3 | LST19 |
| 112 | 20C | A | buffalo | B1 | C2 | 6 | 5 | 5 | 3 | LST8 |
| 113 | 21C | B | buffalo | B1 | C2 | 6 | 5 | 5 | 3 | LST8 |
| 114 | 22C | B | buffalo | B1 | C2 | 6 | 5 | 5 | 3 | LST8 |
| 115 | 23C | B | buffalo | B1 | C2 | 6 | 5 | 5 | 3 | LST8 |
| 116 | 24C | B | cattle | B1 | C1 | 11 | 5 | 5 | 3 | LST19 |
| 117 | 25C | B | cattle | B1 | C2 | 6 | 5 | 5 | 3 | LST8 |

**Additional Table 1** Continued.

| **No** | **Strain code** | **Capsular type** | **Animal host** | **DNA cluster** | **Protein cluster** | **Loop 1 type** | **Loop 2 type** | **Loop 3 type** | **Loop 4 type** | **LST type** |
| --- | --- | --- | --- | --- | --- | --- | --- | --- | --- | --- |
| 118 | 26C | B | buffalo | B1 | C1 | 11 | 5 | 5 | 3 | LST19 |
| 119 | 27C | B | buffalo | B1 | C2 | 6 | 5 | 5 | 3 | LST8 |
| 120 | 28C | B | cattle | B1 | C1 | 11 | 5 | 5 | 3 | LST19 |
| 121 | 29C | B | NP | B1 | C2 | 6 | 5 | 5 | 3 | LST8 |
| 122 | 32C | B | buffalo | B1 | C1 | 11 | 5 | 5 | 3 | LST19 |
| 123 | 33C | B | buffalo | B1 | C1 | 11 | 5 | 5 | 3 | LST19 |
| 124 | 34C | B | buffalo | B1 | C1 | 11 | 5 | 5 | 3 | LST19 |
| 125 | 35C | B | buffalo | B1 | C1 | 11 | 5 | 5 | 3 | LST19 |
| 126 | 36C | B | buffalo | B1 | C1 | 11 | 5 | 5 | 3 | LST19 |
| 127 | 37C | B | buffalo | B1 | C2 | 6 | 5 | 5 | 3 | LST8 |
| 128 | 38C | B | buffalo | B1 | C1 | 11 | 5 | 5 | 3 | LST19 |
| 129 | 39C | B | cattle | B1 | C1 | 11 | 5 | 5 | 3 | LST19 |
| 130 | 40C | B | NP | B1 | C2 | 6 | 5 | 5 | 3 | LST8 |
| 131 | 41C | B | buffalo | B1 | C1 | 11 | 5 | 5 | 3 | LST19 |
| 132 | 42C | B | cattle | B1 | C2 | 6 | 5 | 5 | 3 | LST8 |

**Additional Table 1** Continued.

| **No** | **Strain code** | **Capsular type** | **Animal host** | **DNA cluster** | **Protein cluster** | **Loop 1 type** | **Loop 2 type** | **Loop 3 type** | **Loop 4 type** | **LST type** |
| --- | --- | --- | --- | --- | --- | --- | --- | --- | --- | --- |
| 133 | 43C | B | cattle | B1 | C2 | 6 | 5 | 5 | 3 | LST8 |
| 134 | 44C | B | buffalo | B1 | C2 | 6 | 5 | 5 | 3 | LST8 |
| 135 | 45C | B | buffalo | B1 | C1 | 11 | 5 | 5 | 3 | LST19 |
| 136 | 46C | B | buffalo | B1 | C1 | 11 | 5 | 5 | 3 | LST19 |
| 137 | 47C | B | buffalo | B1 | C2 | 6 | 5 | 5 | 3 | LST8 |
| 138 | 48C | B | buffalo | B1 | C2 | 6 | 5 | 5 | 3 | LST8 |
| 139 | 49C | B | buffalo | B1 | C2 | 6 | 5 | 5 | 3 | LST8 |
| 140 | 50C | B | buffalo | B1 | C2 | 6 | 5 | 5 | 3 | LST8 |
| 141 | 51C | B | buffalo | B1 | C2 | 6 | 5 | 5 | 3 | LST8 |
| 142 | 52C | B | NP | B1 | C2 | 6 | 5 | 5 | 3 | LST8 |
| 143 | 53C | B | NP | B1 | C2 | 6 | 5 | 5 | 3 | LST8 |
| 144 | 54C | B | buffalo | B1 | C2 | 6 | 5 | 5 | 3 | LST8 |
| 145 | 55C | B | buffalo | B1 | C1 | 11 | 5 | 5 | 3 | LST19 |
| 146 | 56C | B | buffalo | B1 | C1 | 11 | 5 | 5 | 3 | LST19 |
| 147 | 57C | B | buffalo | B1 | C2 | 6 | 5 | 5 | 3 | LST8 |

**Additional Table 1** Continued.

| **No** | **Strain code** | **Capsular type** | **Animal host** | **DNA cluster** | **Protein cluster** | **Loop 1 type** | **Loop 2 type** | **Loop 3 type** | **Loop 4 type** | **LST type** |
| --- | --- | --- | --- | --- | --- | --- | --- | --- | --- | --- |
| 148 | 58C | B | buffalo | B1 | C1 | 11 | 5 | 5 | 3 | LST19 |
| 149 | 59C | B | buffalo | B1 | C2 | 6 | 5 | 5 | 3 | LST8 |
| 150 | 60C | B | cattle | B1 | C2 | 6 | 5 | 5 | 3 | LST8 |
| 151 | 61C | B | cattle | B1 | C2 | 6 | 5 | 5 | 3 | LST8 |
| 152 | 62C | B | buffalo | B1 | C2 | 6 | 5 | 5 | 3 | LST8 |
| 153 | 63C | B | buffalo | B1 | C2 | 6 | 5 | 5 | 3 | LST8 |
| 154 | 64C | B | buffalo | B1 | C2 | 6 | 5 | 5 | 3 | LST8 |
| 155 | 65C | B | buffalo | B1 | C2 | 6 | 5 | 5 | 3 | LST8 |
| 156 | 66C | B | buffalo | B1 | C2 | 6 | 5 | 5 | 3 | LST8 |
| 157 | 67C | B | buffalo | B1 | C2 | 6 | 5 | 5 | 3 | LST8 |
| 158 | 68C | B | cattle | B1 | C2 | 6 | 5 | 5 | 3 | LST8 |
| 159 | 71C | B | cattle | B1 | C2 | 6 | 5 | 5 | 3 | LST8 |
| 160 | 72C | B | buffalo | B1 | C2 | 6 | 5 | 5 | 3 | LST8 |
| 161 | 73C | B | buffalo | B1 | C2 | 6 | 5 | 5 | 3 | LST8 |
| 162 | 74C | B | buffalo | B1 | C2 | 6 | 5 | 5 | 3 | LST8 |

**Additional Table 1** Continued.

| **No** | **Strain code** | **Capsular type** | **Animal host** | **DNA cluster** | **Protein cluster** | **Loop 1 type** | **Loop 2 type** | **Loop 3 type** | **Loop 4 type** | **LST type** |
| --- | --- | --- | --- | --- | --- | --- | --- | --- | --- | --- |
| 163 | 75C | B | buffalo | B1 | C2 | 6 | 5 | 5 | 3 | LST8 |
| 164 | 76C | B | buffalo | B1 | C2 | 6 | 5 | 5 | 3 | LST8 |
| 165 | 79C | B | buffalo | B1 | C2 | 6 | 5 | 5 | 3 | LST8 |
| 166 | 80C | B | buffalo | B1 | C2 | 6 | 5 | 5 | 3 | LST8 |
| 167 | 81C | B | buffalo | B1 | C2 | 6 | 5 | 5 | 3 | LST8 |
| 168 | 82C | B | buffalo | B1 | C2 | 6 | 5 | 5 | 3 | LST8 |
| 169 | 83C | B | buffalo | B1 | C2 | 6 | 5 | 5 | 3 | LST8 |
| 170 | 84C | B | buffalo | B1 | C2 | 6 | 5 | 5 | 3 | LST8 |
| 171 | 85C | B | buffalo | B1 | C2 | 6 | 5 | 5 | 3 | LST8 |
| 172 | 86C | B | buffalo | B1 | C2 | 6 | 5 | 5 | 3 | LST8 |
| 173 | 87C | B | buffalo | B1 | C2 | 6 | 5 | 5 | 3 | LST8 |
| 174 | 89C | B | buffalo | B1 | C2 | 6 | 5 | 5 | 3 | LST8 |
| 175 | 90C | B | buffalo | B1 | C2 | 6 | 5 | 10 | 3 | LST10 |
| 176 | 91C | B | buffalo | B1 | C2 | 6 | 5 | 10 | 3 | LST10 |
| 177 | 92C | B | buffalo | B1 | C2 | 6 | 5 | 5 | 3 | LST8 |

**Additional Table 1** Continued.

| **No** | **Strain code** | **Capsular type** | **Animal host** | **DNA cluster** | **Protein cluster** | **Loop 1 type** | **Loop 2 type** | **Loop 3 type** | **Loop 4 type** | **LST type** |
| --- | --- | --- | --- | --- | --- | --- | --- | --- | --- | --- |
| 178 | 93C | B | cattle | B1 | C2 | 6 | 5 | 5 | 3 | LST8 |
| 179 | 94C | B | cattle | B1 | C2 | 6 | 5 | 10 | 3 | LST10 |
| 180 | 95C | B | cattle | B1 | C2 | 6 | 5 | 5 | 3 | LST8 |
| 181 | 96C | B | NP | B1 | C2 | 6 | 5 | 5 | 3 | LST8 |
| 182 | 97C | B | NP | B1 | C2 | 6 | 5 | 5 | 3 | LST8 |
| 183 | 98C | B | NP | B1 | C2 | 6 | 5 | 5 | 3 | LST8 |
| 184 | 99C | B | NP | B1 | C2 | 6 | 5 | 5 | 3 | LST8 |
| 185 | 100C | B | NP | B1 | C2 | 6 | 5 | 5 | 3 | LST8 |
| 186 | 101C | B | NP | B1 | C2 | 6 | 5 | 5 | 3 | LST8 |
| 187 | PMstr_HN06 | D | pig | P3 | NP | 10 | 2 | 2 | 3 | LST18 |
| 188 | PMstr_Pm70 | A | chicken | P4 | NP | 2 | 2 | 2 | 2 | LST2 |
| 189 | PMstr_43A | NP | NP | NP | B1 | 15 | 9 | 11 | 9 | LST24 |
| 190 | PM_36950 | NP | Bovine | NP | NP | 4 | 1 | 1 | 1 | LST5 |
| 191 | PM_str_OB_47B | NP | NP | C* | B1 | 6 | 6 | 5 | 3 | LST9 |
| 192 | Clone_yak | B | Yak | C2 | B1 | 6 | 5 | 5 | 3 | LST8 |

**Additional Table 1** Continued.

| **No** | **Strain code** | **Capsular type** | **Animal host** | **DNA cluster** | **Protein cluster** | **Loop 1 type** | **Loop 2 type** | **Loop 3 type** | **Loop 4 type** | **LST type** |
| --- | --- | --- | --- | --- | --- | --- | --- | --- | --- | --- |
| 193 | PN_11B | NP | NP | C2 | B1 | 6 | 5 | 5 | 3 | LST8 |
| 194 | PN_13B | NP | NP | C2 | B1 | 6 | 5 | 5 | 3 | LST8 |
| 195 | OB_34B | NP | NP | C2 | B1 | 6 | 5 | 5 | 3 | LST8 |
| 196 | OB_26A | NP | NP | C2 | B1 | 6 | 5 | 5 | 3 | LST8 |
| 197 | OB_17B | NP | NP | C2 | B1 | 6 | 5 | 5 | 3 | LST8 |
| 198 | OB_37B | NP | NP | C2 | B1 | 6 | 5 | 5 | 3 | LST8 |
| 199 | OB_24A | NP | NP | C2 | B1 | 6 | 5 | 5 | 3 | LST8 |
| 200 | P52 | B | NP | C2 | B1 | 6 | 5 | 5 | 3 | LST8 |
| 201 | OB_3B | NP | NP | C2 | B1 | 7 | 5 | 5 | 3 | LST11 |
| 202 | OB_52A | NP | NP | C2 | B1 | 6 | 5 | 5 | 3 | LST8 |
| 203 | PN_21A | NP | NP | C2 | B1 | 6 | 5 | 5 | 3 | LST8 |
| 204 | PN_15A | NP | NP | C2 | B1 | 6 | 5 | 5 | 3 | LST8 |
| 205 | OB_8A | NP | NP | C2 | B1 | 6 | 5 | 5 | 3 | LST8 |
| 206 | CVCC393 | E | Bovine | C* | B1 | 16 | 10 | 12 | 10 | LST25 |
| 207 | OB_50A | NP | NP | C* | B1 | 17 | 10 | 12 | 10 | LST26 |

**Additional Table 1** Continued.

| **No** | **Strain code** | **Capsular type** | **Animal host** | **DNA cluster** | **Protein cluster** | **Loop 1 type** | **Loop 2 type** | **Loop 3 type** | **Loop 4 type** | **LST type** |
| --- | --- | --- | --- | --- | --- | --- | --- | --- | --- | --- |
| 208 | 32B | NP | NP | P4 | P4I | 14 | 2 | 2 | 8 | LST23 |
| 209 | 95120769 | NP | Bovine | P1 | P1 | 3 | 3 | 3 | 3 | LST4 |
| 210 | Clone_xj | NP | goose | P4 | P4I | 5 | 4 | 4 | 4 | LST7 |
| 211 | T931317 | NP | Bovine | P4 | P4I | 2 | 4 | 4 | 4 | LST3 |
| 212 | IndPm113 | NP | Sheep | P2 | P2 | 12 | 11 | 13 | 3 | LST21 |
| 213 | T94289 | NP | Bovine | P4 | P4I | 19 | 4 | 4 | 4 | LST28 |
| 214 | 95010872 | NP | Bovine | P4 | P4I | 2 | 2 | 2 | 2 | LST2 |
| 215 | XJNKY_12_YF1 | A | Sheep | NP | P3 | 21 | 1 | 14 | 1 | LST30 |
| 216 | XJNKY_10_2YF1_2 | A | Sheep | NP | P3 | 18 | 1 | 14 | 1 | LST27 |
| 217 | XJNKY_10_2YF1_1 | A | Sheep | NP | P3 | 18 | 1 | 14 | 1 | LST27 |
| 218 | XJ121_ | NP | NP | NP | P3 | 20 | 1 | 1 | 1 | LST29 |
| 219 | Clone_121_3 | A | Bovine | NP | P3 | 4 | 1 | 1 | 1 | LST5 |
| 220 | 39A | NP | NP | NP | P3 | 1 | 1 | 1 | 1 | LST1 |
| 221 | 27A | NP | NP | NP | P3 | 1 | 1 | 1 | 1 | LST1 |
| 222 | 38A | NP | NP | NP | P3 | 1 | 1 | 1 | 1 | LST1 |

**Additional Table 1** Continued.

| **No** | **Strain code** | **Capsular type** | **Animal host** | **DNA cluster** | **Protein cluster** | **Loop 1 type** | **Loop 2 type** | **Loop 3 type** | **Loop 4 type** | **LST type** |
| --- | --- | --- | --- | --- | --- | --- | --- | --- | --- | --- |
| 223 | 22B | NP | NP | NP | P3 | 1 | 1 | 1 | 1 | LST1 |
| 224 | 49A | NP | NP | NP | P3 | 1 | 1 | 1 | 1 | LST1 |
| 225 | P70 | A | Bovine | NP | P3 | 4 | 1 | 1 | 7 | LST6 |
| 226 | 20B | NP | NP | NP | P3 | 1 | 1 | 1 | 1 | LST1 |
| 227 | 96020298 | NP | Bovine | NP | P3 | 4 | 1 | 1 | 1 | LST5 |
