# Supplementary material for: OmpA protein sequence-based typing and virulence-associated gene profiles of Pasteurella multocida isolates associated with bovine haemorrhagic septicaemia and porcine pneumonic pasteurellosis in Thailand
Source: BMC Vet Res. 2017 Aug 16;13:243. doi: 10.1186/s12917-017-1157-6 (PMC5559837; doi:10.1186/s12917-017-1157-6)
Supplement: Supplementary file 2 — Information of 191 P. multocida isolates associated with diseased pigs, cattle and water buffaloes in Thailand collected from 1989 to 2012. These isolates were collected and maintained by the National Institute of Animal Health, Department of Livestock, Ministry of Agriculture and Cooperatives. Each isolate was named with two-letter code. (DOCX 38 kb) [file 12917_2017_1157_MOESM2_ESM.docx]

**Additional file 2: Table S2** Information of 191 *P. multocida* isolates associated with diseased pigs, cattle and water buffaloes in Thailand collected from 1989 to 2012. These isolates were collected and maintained by the National Institute of Animal Health, Department of Livestock, Ministry of Agriculture and Cooperatives. Each isolate was named with two-letter code.

| **No** | **Isolate code** | **Capsular type** | **Year** | **Animal host** | **Organ** |
| --- | --- | --- | --- | --- | --- |
| 1 | 1P | A | 2006 | pig | lung |
| 2 | 2P | D | 2006 | pig | lung |
| 3 | 3P | D | 2006 | pig | brain |
| 4 | 5P | D | 2006 | pig | lung |
| 5 | 6P | A | 2006 | pig | NP |
| 6 | 7P | D | 2006 | pig | lung |
| 7 | 8P | A | 2006 | pig | lung |
| 8 | 9P | A | 2006 | pig | lung |
| 9 | 10P | A | 2006 | pig | lung |
| 10 | 11P | A | 2006 | pig | lymph node |
| 11 | 12P | A | 2006 | pig | lung |
| 12 | 13P | A | 2006 | pig | lung |
| 13 | 14P | A | 2007 | pig | lung |
| 14 | 15P | A | 2007 | pig | lung |
| 15 | 16P | A | 2007 | pig | lung |
| 16 | 17P | D | 2007 | pig | lung |
| 17 | 18P | A | 2007 | pig | lung |
| 18 | 20P | D | 2007 | pig | lung |
| 19 | 21P | A | 2007 | pig | lung |
| 20 | 23P | D | 2007 | pig | heart |
| 21 | 24P | A | 2007 | pig | lung |
| 22 | 25P | A | 2007 | pig | lung |
| 23 | 26P | A | 2007 | pig | lung |
| 24 | 27P | A | 2007 | pig | lung |
| 25 | 28P | A | 2007 | pig | lung |
| 26 | 29P | A | 2007 | pig | lung |
| 27 | 30P | A | 2007 | pig | lung |
| 28 | 31P | A | 2007 | pig | lung |

**Additional Table 2** Continued.

| **No** | **Isolate code** | **Capsular type** | **Year** | **Animal host** | **Organ** |
| --- | --- | --- | --- | --- | --- |
| 29 | 32P | A | 2007 | pig | lung |
| 30 | 33P | A | 2007 | pig | lung |
| 31 | 34P | A | 2007 | pig | lung |
| 32 | 35P | A | 2007 | pig | lung |
| 33 | 36P | A | 2007 | pig | lung |
| 34 | 37P | D | 2007 | pig | heart |
| 35 | 38P | D | 2007 | pig | lung |
| 36 | 39P | D | 2007 | pig | liver |
| 37 | 40P | D | 2007 | pig | kidney |
| 38 | 41P | D | 2007 | pig | heart |
| 39 | 42P | D | 2007 | pig | kidney |
| 40 | 43P | D | 2007 | pig | heart |
| 41 | 44P | A | 2007 | pig | lung |
| 42 | 47P | A | 2007 | pig | lung |
| 43 | 48P | A | 2007 | pig | lung |
| 44 | 49P | A | 2007 | pig | lung |
| 45 | 50P | A | 2007 | pig | lung |
| 46 | 51P | A | 2007 | pig | lung |
| 47 | 52P | A | 2008 | pig | lung |
| 48 | 53P | A | 2008 | pig | lung |
| 49 | 55P | D | 2008 | pig | lung |
| 50 | 58P | A | 2008 | pig | lung |
| 51 | 59P | A | 2008 | pig | lung |
| 52 | 60P | A | 2008 | pig | lung |
| 53 | 62P | A | 2008 | pig | NP |
| 54 | 63P | A | 2008 | pig | lung |
| 55 | 65P | A | 2008 | pig | lung |
| 56 | 66P | A | 2008 | pig | lung |
| 57 | 67P | A | 2008 | pig | lung |
| 58 | 68P | A | 2008 | pig | tonsil |
| 59 | 69P | A | 2008 | pig | plate culture |
| 60 | 70P | A | 2008 | pig | plate culture |

**Additional Table 2** Continued.

| **No** | **Isolate code** | **Capsular type** | **Year** | **Animal host** | **Organ** |
| --- | --- | --- | --- | --- | --- |
| 61 | 71P | A | 2008 | pig | plate culture |
| 62 | 72P | A | 2008 | pig | plate culture |
| 63 | 73P | A | 2008 | pig | lung |
| 64 | 74P | A | 2008 | pig | lung |
| 65 | 78P | A | 2008 | pig | lung |
| 66 | 79P | A | 2008 | pig | lung |
| 67 | 80P | A | 2008 | pig | lung |
| 68 | 81P | A | 2008 | pig | tonsil |
| 69 | 82P | D | 2008 | pig | lung |
| 70 | 83P | A | 2008 | pig | lung |
| 71 | 84P | A | 2008 | pig | NP |
| 72 | 85P | D | 2008 | pig | lung |
| 73 | 86P | A | 2008 | pig | lung |
| 74 | 87P | D | 2009 | pig | empyema |
| 75 | 88P | D | 2009 | pig | lung |
| 76 | 89P | A | 2009 | pig | lung |
| 77 | 90P | A | 2009 | pig | lung |
| 78 | 91P | A | 2009 | pig | lung |
| 79 | 92P | A | 2009 | pig | lung |
| 80 | 93P | D | 2009 | pig | lung |
| 81 | 94P | A | 2009 | pig | lung |
| 82 | 95P | A | 2009 | pig | lung |
| 83 | 96P | D | 2009 | pig | lung |
| 84 | 97P | A | 2009 | pig | liver |
| 85 | 98P | A | 2009 | pig | lung |
| 86 | 99P | A | 2009 | pig | lung |
| 87 | 100P | A | 2009 | pig | lung |
| 88 | 101P | A | 2009 | pig | lung |
| 89 | 102P | A | 2009 | pig | lung |
| 90 | 103P | A | 2009 | pig | lung |
| 91 | 104P | D | 2009 | pig | lung |
| 92 | 108P | A | 2009 | pig | lung |

**Additional Table 2** Continued.

| **No** | **Isolate code** | **Capsular type** | **Year** | **Animal host** | **Organ** |
| --- | --- | --- | --- | --- | --- |
| 93 | 1C | A | 1989 | buffalo | NP |
| 94 | 2C | B | 1989 | buffalo | NP |
| 95 | 3C | B | 1989 | buffalo | NP |
| 96 | 4C | B | 1989 | buffalo | NP |
| 97 | 5C | B | 1989 | buffalo | NP |
| 98 | 6C | B | 1989 | buffalo | NP |
| 99 | 7C | B | 1990 | cattle | NP |
| 100 | 8C | B | 1992 | buffalo | NP |
| 101 | 9C | B | 1993 | cattle | NP |
| 102 | 10C | B | 1994 | buffalo | NP |
| 103 | 11C | B | 1994 | buffalo | NP |
| 104 | 12C | A | 1995 | cattle | NP |
| 105 | 13C | B | 1995 | buffalo | lung |
| 106 | 14C | B | 1996 | buffalo | NP |
| 107 | 15C | B | 1996 | buffalo | NP |
| 108 | 16C | B | 1996 | buffalo | NP |
| 109 | 17C | B | 1996 | buffalo | NP |
| 110 | 18C | B | 1997 | buffalo | NP |
| 111 | 19C | B | 1997 | buffalo | heart |
| 112 | 20C | A | 1997 | buffalo | NP |
| 113 | 21C | B | 1998 | buffalo | NP |
| 114 | 22C | B | 1998 | buffalo | NP |
| 115 | 23C | B | 2000 | buffalo | NP |
| 116 | 24C | B | 2000 | cattle | NP |
| 117 | 25C | B | 2000 | cattle | NP |
| 118 | 26C | B | 2001 | buffalo | NP |
| 119 | 27C | B | 2001 | buffalo | NP |
| 120 | 28C | B | 2001 | cattle | NP |
| 121 | 29C | B | NP | NP | NP |
| 122 | 30C | B | 2001 | cattle | heart |
| 123 | 32C | B | 2001 | buffalo | lung |

**Additional Table 2** Continued.

| **No** | **Isolate code** | **Capsular type** | **Year** | **Animal host** | **Organ** |
| --- | --- | --- | --- | --- | --- |
| 124 | 33C | B | 2001 | buffalo | liver |
| 125 | 34C | B | 2001 | buffalo | heart |
| 126 | 35C | B | 2001 | buffalo | NP |
| 127 | 36C | B | 2001 | buffalo | NP |
| 128 | 37C | B | 2001 | buffalo | NP |
| 129 | 38C | B | 2002 | buffalo | NP |
| 130 | 39C | B | 2002 | cattle | NP |
| 131 | 40C | B | NP | NP | NP |
| 132 | 41C | B | 2003 | buffalo | NP |
| 133 | 42C | B | 2003 | cattle | NP |
| 134 | 43C | B | 2003 | cattle | NP |
| 135 | 44C | B | 2003 | buffalo | NP |
| 136 | 45C | B | 2003 | buffalo | NP |
| 137 | 46C | B | 2003 | buffalo | lung |
| 138 | 47C | B | 2004 | buffalo | NP |
| 139 | 48C | B | 2004 | buffalo | NP |
| 140 | 49C | B | 2005 | buffalo | NP |
| 141 | 50C | B | 2005 | buffalo | NP |
| 142 | 51C | B | 2006 | buffalo | NP |
| 143 | 52C | B | NP | NP | NP |
| 144 | 53C | B | NP | NP | NP |
| 145 | 54C | B | 2006 | buffalo | NP |
| 146 | 55C | B | 2006 | buffalo | heart |
| 147 | 56C | B | 2006 | buffalo | lung |
| 148 | 57C | B | 2006 | buffalo | NP |
| 149 | 58C | B | 2006 | buffalo | NP |
| 150 | 59C | B | 2007 | buffalo | NP |
| 151 | 60C | B | 2008 | cattle | liver |
| 152 | 61C | B | 2008 | cattle | kidney |
| 153 | 62C | B | 2008 | buffalo | liver |
| 154 | 63C | B | 2008 | buffalo | lung |

**Additional Table 2** Continued.

| **No** | **Isolate code** | **Capsular type** | **Year** | **Animal host** | **Organ** |
| --- | --- | --- | --- | --- | --- |
| 155 | 64C | B | 2008 | buffalo | NP |
| 156 | 65C | B | 2008 | buffalo | heart |
| 157 | 66C | B | 2008 | buffalo | blood |
| 158 | 67C | B | 2008 | buffalo | liver |
| 159 | 68C | B | 2008 | cattle | lung |
| 160 | 69C | B | 2008 | buffalo | liver |
| 161 | 70C | B | 2008 | buffalo | NP |
| 162 | 71C | B | 2008 | cattle | NP |
| 163 | 72C | B | 2008 | buffalo | lung |
| 164 | 73C | B | 2008 | buffalo | lung |
| 165 | 74C | B | 2008 | buffalo | liver |
| 166 | 75C | B | 2009 | buffalo | NP |
| 167 | 76C | B | 2009 | buffalo | lymph node |
| 168 | 77C | B | 2009 | buffalo | heart |
| 169 | 78C | B | 2009 | buffalo | lymph node |
| 170 | 79C | B | 2009 | buffalo | NP |
| 171 | 80C | B | 2009 | buffalo | NP |
| 172 | 81C | B | 2009 | buffalo | NP |
| 173 | 82C | B | 2009 | buffalo | NP |
| 174 | 83C | B | 2009 | buffalo | NP |
| 175 | 84C | B | 2009 | buffalo | liver |
| 176 | 85C | B | 2009 | buffalo | lung |
| 177 | 86C | B | 2008 | buffalo | heart |
| 178 | 87C | B | 2008 | buffalo | NP |
| 179 | 89C | B | 2010 | buffalo | NP |
| 180 | 90C | B | 2011 | buffalo | NP |
| 181 | 91C | B | 2011 | buffalo | NP |
| 182 | 92C | B | 2011 | buffalo | NP |
| 183 | 93C | B | 2011 | cattle | NP |
| 184 | 94C | B | 2011 | cattle | NP |
| 185 | 95C | B | 2012 | cattle | NP |
| 186 | 96C | B | 2000 | NP | NP |

**Additional Table 2** Continued.

| **No** | **Isolate code** | **Capsular type** | **Year** | **Animal host** | **Organ** |
| --- | --- | --- | --- | --- | --- |
| 187 | 97C | B | NP | NP | NP |
| 188 | 98C | B | NP | NP | NP |
| 189 | 99C | B | NP | NP | NP |
| 190 | 100C | B | NP | NP | NP |
| 191 | 101C | B | NP | NP | NP |

Note: NP: information not available
